# Traditional Chinese medicine prescription Guizhi Fuling Pills ameliorate cisplatin-induced renal injury via remodeling intestinal homeostasis in mice with tongue squamous cell carcinoma

**DOI:** 10.3389/fphar.2025.1631966

**Published:** 2025-10-02

**Authors:** Kai Fu, Shaoning Yin, Tengfei Liu, Weiyi Wang

**Affiliations:** ^1^ Department of Otolaryngology Head and Neck Surgery, The Fourth Hospital of Hebei Medical University, Shijiazhuang, Hebei, China; ^2^ Department of Hematology, The Fourth Hospital of Hebei Medical University, Shijiazhuang, Hebei, China; ^3^ Department of General Surgery, Xingtai People’s Hospital, Xingtai, Hebei, China; ^4^ Department of Immunology and Pathobiology, Hebei University of Chinese Medicine, Shijiazhuang, Hebei, China

**Keywords:** Guizhi Fuling Pills, cisplatin, renal injury, gut homeostasis, tongue squamous cell carcinoma

## Abstract

**Introduction:**

Guizhi Fuling Pills, a traditional Chinese medicine, may affect cisplatin-induced kidney damage during tongue squamous cell carcinoma (TSCC) treatment via the gut–kidney axis, but this is not well understood. This study explores the impact of Guizhi Fuling Pills on gut microbiota and intestinal barrier function, its protective effects against cisplatin-induced kidney injury, and the mechanisms involved.

**Methods:**

A tongue tumor model was created in nude mice using Cal27 cells. Treatments included cisplatin, Guizhi Fuling Pills, or both. Kidney function was evaluated through serum creatinine (CRE) and blood urea nitrogen (BUN). Oxidative stress and inflammation were assessed by measuring malondialdehyde (MDA), superoxide dismutase (SOD) activity, and tumor necrosis factor-α (TNF-α). Gut microbiota was analyzed via 16S rRNA sequencing, and intestinal morphology was examined using H&E and PAS staining.

**Results:**

The coadministration of Guizhi Fuling Pills with DDP demonstrated multifaceted effects. At the systemic level, it ameliorated renal pathological changes, as evidenced by decreased serum levels of CRE, BUN, MDA, and TNF-α, alongside increased SOD activity. It also enhanced the inhibitory effect on TSCC tumor growth. In the gut, this combination reduced crypt depth, increased goblet cell number, and favorably modulated the microbiota by decreasing the abundance of Desulfovibrioand increasing beneficial bacteria including Lactobacillus, Kineothrix, and Eubacterium, without affecting ZO-1 expression.

**Discussion:**

Guizhi Fuling Pill effectively mitigates cisplatin-induced renal injury and maintains its anti-tumor efficacy in TSCC by modulating gut microflora and reducing inflammation.

## 1 Introduction

Tongue squamous cell carcinoma (TSCC) represents a common and aggressive malignancy within the oral cavity, characterized by a persistently increasing global incidence. Despite advances in treatment strategies, the 5-year survival rate for patients with TSCC remains unsatisfactory. Cisplatin (DDP) is one of the most frequently used chemotherapeutic agents for the treatment of TSCC; however, its clinical utility is substantially limited by dose-dependent toxicities, particularly nephrotoxicity (Yuan et al., 2024). Guizhi Fuling Pills (GZFLs), a classical traditional Chinese medicine formulation, are known for their properties of promoting blood circulation, resolving blood stasis, and dissipating abdominal masses.

Clinically, Guizhi Fuling Pills are used in the treatment of uterine fibroids and ovarian cancer (Chen et al., 2014; Wang et al., 2022), with reduced toxicity and side effects reported. DDP therapy is associated with a high incidence of nephrotoxicity, affecting approximately one-third of treated patients (Pabla and Dong, 2008). The dose-dependent and cumulative nature of this nephrotoxicity frequently necessitates dose reduction or treatment discontinuation, although these interventions often attenuate antitumor efficacy (Latcha et al., 2016). DDP-induced nephrotoxicity primarily arises from acute renal excretory dysfunction, resulting in the accumulation of serum metabolic wastes such as blood urea nitrogen (BUN) and creatinine (CRE) (Shahid et al., 2018). An alternative mechanism proposes that DDP induces gut microbiota dysbiosis, which subsequently contributes to renal dysfunction. Consequently, targeting the gut–kidney axis represents a promising strategy for mitigating cisplatin-induced nephrotoxicity (McSweeney et al., 2021; Gharaie et al., 2023). In clinical contexts, the gut microbiota is regarded as a critical modulator of the gut–kidney axis and plays an indispensable role in the maintenance of renal homeostasis. Modulation of the gut microbiota has been applied in the treatment of various diseases. *Desulfovibrio*, a harmful intestinal bacterium, causes intestinal damage (Han et al., 2021). Emerging evidence indicates that gut microbiota modulation through the gut–kidney axis plays a protective role in alleviating renal injury. Decreased production of short-chain fatty acids (SCFAs) exacerbates renal injury, whereas beneficial bacteria such as Kineothrix and *Lactobacillus* contribute to SCFA production (Liu W. et al., 2024; Li et al., 2020). Notably, SCFAs are critically involved in mitigating renal pathology (Xu et al., 2022). At the same time, certain pharmacological agents have been reported to alleviate DDP-induced renal injury through the enterorenal axis (Tian et al., 2024). However, it remains unknown whether Guizhi Fuling Pills attenuate cisplatin nephrotoxicity via modulation of the gut–kidney axis (Tian et al., 2024). Therefore, we determined whether the nephroprotective effects of Guizhi Fuling Pills against cisplatin-induced renal injury are associated with modulation of gut microbiota composition and intestinal barrier function.

## 2 Materials and methods

### 2.1 Preparation of Guizhi Fuling Pills

Guizhi Fuling Pills (batch number: 123004) were purchased from Shanxi Tiansheng Pharmaceutical Co., Ltd (Shanxi, China). sealed, and stored at room temperature in the Immunology and Pathobiology Laboratory of Hebei University of Chinese Medicine. The production date is 31 January 2024, and the validity period extends until December 2027. Its composition is presented in Table 1. Five kinds of raw materials were crushed into powder, then sieved and mixed evenly, and subsequently combined with refined honey (100 g) to prepare large honey pills. The quality standard of Guizhi Fuling Pills (SFDA approval number: Z14020749) was assessed according to guidelines on page 1439 in the Chinese Pharmacopoeia (2020 edition). Sample preparation for animal administration was as follows: Guizhi Fuling Pills (13.104 g) were immersed in 42 mL of purified water and subjected to ultrasonic dissolution (360 W, 10 min × 3) at room temperature to obtain a homogeneous aqueous solution. The solution was subsequently filtered through a 0.22-μm membrane, yielding a final concentration of 0.312 g/mL, and stored at 4 °C until further use. The dosing solution was prepared fresh daily, administered within a 2-h window following preparation.

**TABLE 1:** The composition of Guizhi Fuling Pills (GZFL).

NO.	Chinese name	Latin name	Family	Part used	Processing method	Weight (g)
1	Guizhi	*Cinnamomi Ramulus*	Lauraceae	Branch	Purify, wash, soften, slice thickly, dry at low temperature, and powder	20
2	Fuling	Poria	Polyporaceae	Sclerotium	Soak, wash, steam briefly, peel, slice thickly, dry, and powder	20
3	Taoren	Persicae Semen	Rosaceae	Seed	Remove impurities, dry, and crush into fine powder	20
4	Chishao	Paeoniae Radix Rubra	Ranunculaceae	Root	Remove impurities, wash, size-separate, soften, slice, dry, and powder	20
5	Mudanpi	Moutan Cortex	Ranunculaceae	Root	Quick wash, moisten, slice thinly, sun-dry, and pulverize	20

### 2.2 UPLC-MS

Sample preparation for UPLC-MS analysis: The sample for UPLC-MS analysis was prepared from Guizhi Fuling Pills. First, to simulate the oral administration form, 13.104 g of the pills was immersed in 42 mL of purified water and subjected to ultrasonic dissolution (360 W, 10 min × 3) at room temperature. The solution was filtered through a 0.22-μm membrane, yielding a stock solution of 0.312 g/mL, and stored at 4 °C for further analysis.

UPLC-MS analyses were performed using a Vanquish liquid chromatography system coupled with an Orbitrap Exploris 120 mass spectrometer. The prepared solution of Guizhi Fuling Pills was centrifuged at 4 °C and 12,000 rpm for 15 min, and 300 μL of the supernatant was pipetted into a new container, followed by the addition of 100 μL of the extract (methanol: acetonitrile: water in a ratio of 2:2:1). The mixed solutions were vortexed for 30 s, sonicated for 5 min in a 4 °C water bath, and incubated for 1 h at −20 °C to precipitate proteins. The mixture was then centrifuged at 4 °C and 12,000 rpm for 15 min; the supernatant was filtered through a 0.22-μm filter into an injection vial for analysis. The target compounds were separated using a Phenomenex Kinetex C18 (2.1 mm × 100 mm, 2.6 μm) liquid (Thermo Fisher Scientific, California, United States) ultra-high-performance liquid chromatograph system. In liquid chromatography, the A phase was water, containing 0.01% acetic acid, and the B phase was isopropanol:acetonitrile (1:1). The sample plate temperature was maintained at 4 °C, and the injection volume was 2 μL. An Orbitrap Exploris 120 mass spectrometer (Thermo Fisher Scientific) was used for mass spectrometry analysis, and chromatographic data were collected in negative ionization and positive ionization modes. The ESI source conditions were set as follows: sheath gas flow rate 50 Arb; Aux gas flow rate 15 Arb; capillary temperature 320 °C; full MS resolution 60,000; MS/MS resolution 15,000; collision energy SNCE 20/30/40; spray voltage 3.8 kV (positive) or 3.4 kV (negative). ProteoWizard software was used to process the original data.

Metabolite analysis was performed using an Orbitrap Exploris 120 mass spectrometer operating in the data-dependent acquisition (DDA) mode to nonselectively capture all detectable ions, encompassing both known and unknown metabolites. Metabolite identification was achieved by matching experimental data against the BiotreeDB (V3.0) database, which contains reference information on retention time (RT), precursor ion mass-to-charge ratio (MS1), and tandem mass spectrometry (MS2) spectra. The identities of metabolites detected in samples were confirmed by matching their MS1, MS2, and RT data with those of authentic chemical standards.

### 2.3 Animals

Male BALB/c-nude mice were purchased from Sibeifu (Beijing) Biotechnology Co., Ltd. and fed in a barrier facility in a specific pathogen-free (SPF) environment, with all mice having free access to food and water. All animal experiments were approved by the Animal Experiment Ethics Committee of Hebei University of Chinese Medicine (No. DWLL202409007).

An orthotopic tongue cancer model was established in nude mice by submucosal injection of 2 × 10^5^ Cal27 cells into the mid-left tongue margin (Zhang et al., 2025). The mice were then randomly assigned to four groups (n = 5 per group): control, DDP, GZFL, and GZFL + DDP.

Mice in the GZFL group received daily oral administration of Guizhi Fuling Pills at 1.56 g/kg for 13 consecutive days. This dose was derived from the human clinical dose (12 g/day for a 70 kg adult) and converted to the mouse equivalent using a standard conversion factor of 9.1. Mice in the DDP group were administered cisplatin (15663-27-1, Sigma, Missouri, United States), dissolved in normal saline, via intraperitoneal injection at 2 mg/kg every other day (Yang et al., 2024). Mice in the control group received daily oral gavage of 0.1 mL purified water for 13 days. Those in the combination (GZFL + DDP) group were administered Guizhi Fuling Pills (1.56 g/kg, daily by gavage) along with cisplatin (2 mg/kg, dissolved in normal saline, injected intraperitoneally every other day) for 13 days. Then, the tumor volume was measured using the formula: V = π/6 × L × W × H (L, tumor length; W, tumor width; H, tumor height).

### 2.4 Cell culture

The human tongue cell carcinoma line Cal27 was obtained from the American Type Culture Collection (ATCC, Manassas, VA, United States). Cells were cultured in high-glucose Dulbecco’s modified Eagle medium (DMEM) supplemented with 10% fetal bovine serum (FBS) and maintained at 37 °C in a humidified atmosphere of 5% CO_2_.

### 2.5 Detection of serum biochemical indices

According to the instructions of the reagent kits, the CRE and BUN contents in each group of serum samples were detected using the CRE reagent kit (C011-2-1, NJJCBIO) and the BUN reagent kit (C011-2-1, NJJCBIO), respectively.

### 2.6 Detection of the renal oxidative damage index

The levels of superoxide dismutase (SOD) and malondialdehyde (MDA) in renal tissue homogenates were measured using commercial assay kits (SOD: A001-1-2; MDA: A003-1-2, NJJCBIO), according to the manufacturer’s instructions.

### 2.7 ELISA experiment

According to the kit instructions, the TNF-α kit (mIC50536-1, Mlbio, Shanghai, China) was used to determine the content of tumor necrosis factor-alpha (TNF-α) in the renal tissue of each group.

### 2.8 16S rRNA sequencing

After 10 days of intervention, feces were collected from each group of mice. The sequencing analysis of mouse microbial diversity was performed by Paisenuo Gene Technology Co., Ltd. (Nanjing, China). We analyzed and plotted species diversity and species differences using the Gene Cloud website (Lu et al., 2024).

### 2.9 Hematoxylin and eosin (HE) staining

The tongue, kidney, and intestine were fixed with 4% paraformaldehyde (BL539A, Biosharp, Hefei, China) and embedded in paraffin. The tongue and kidney were cut into 4-μm sections, and the intestine was cut into 2-μm sections for Hematoxylin and eosin (HE) staining solution (DH0020, LEAGENE, Beijing, China). Tissue morphology was observed, and images were captured using a microscope. Finally, the staining results were analyzed using ImageJ software (National Institutes of Health, NIH).

### 2.10 Periodic acid–Schiff staining (PAS)

Mouse colonic goblet cells were stained using the Periodic acid–Schiff staining (PAS) assay kit (R20526, Yuanye, Shanghai, China), and then, the structure of colonic goblet cells was observed under a microscope. Finally, the staining results were analyzed using ImageJ software (National Institutes of Health, NIH).

### 2.11 Immunohistochemistry (IHC)

The paraffin-embedded colon tissue was cut into 2-μm sections and dewaxed. Afterward, antigen repair solution was added; zonula occludens 1 (ZO-1) (Abcam, Cambridge, United Kingdom, ab276131) was incubated overnight; secondary antibody staining (R20526, Shanghai Yuanye Bio-Technology Co., Ltd) and hematoxylin staining were performed, and the dehydrated slides were observed under a microscope. Finally, the staining results were analyzed using ImageJ software (National Institutes of Health, NIH).

### 2.12 Statistical analysis

Statistics analysis of data was conducted using GraphPad Prism 9.5.1 software (GraphPad Software, United States). The experimental data were described as means ± SD. The 16S data were tested using the Kruskal–Wallis rank sum test and Dunn’s test as a post-test to verify the significance of the difference. Except for 16S data, other data were compared between three groups or more than three groups. One-way analysis of variance (one-way ANOVA) was used to analyze the means between groups. Statistical significance was considered when the P-value was less than 0.05 (Hao et al., 2022).

## 3 Results

### 3.1 Identification of the components of Guizhi Fuling Pills

**FIGURE 1:**
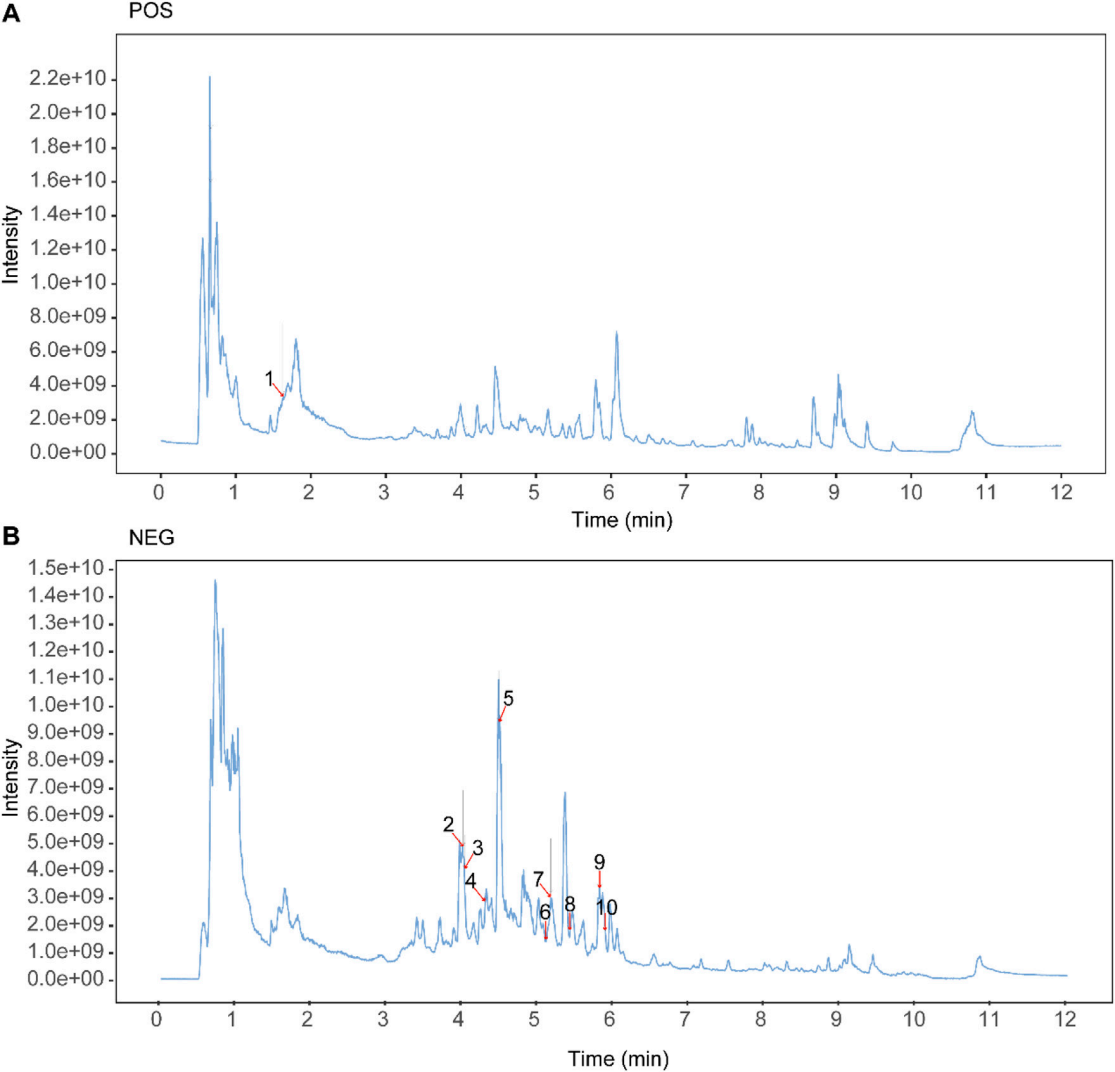
Identification of the components of Guizhi Fuling Pills. **(A)** Total ion current (TIC) chromatogram in the positive ion mode. **(B)** TIC chromatogram in the negative ion mode. The number peak corresponds to the serial number in Table 2. Unlabeled peaks are from known metabolites and other unknown compounds in the public database, and Supplementary Table S1 supplements the relevant information on these unlabeled peaks.

The chemical constituents of Guizhi Fuling Pills were characterized using UPLC-MS/MS. Total ion chromatograms (TICs) of Guizhi Fuling Pills in the positive ion (POS) mode (Figure 1A) and negative ion (NEG) mode (Figure 1B) were obtained. Subsequently, 469 compounds were identified using the BiotreeDB (V3.0) database (Supplementary Table S1). Based on the previous studies on Guizhi Fuling Pills, 10 main compounds were identified, namely, mudanpioside C, propylgallate, amygdalin, catechin, paeoniflorin, benzoic acid, benzoylpaeoniflorin, 3-phenylpropanoic acid, paeonolide, and paeonol (Table 2) (Chen et al., 2009; Zhang et al., 2018). The secondary mass spectra of these ten compounds are provided in the *Supplementary Material*. Amygdalin is a characteristic constituent derived from the Persicae Semen. Catechin is commonly found in both Paeoniae Radix Alba and Moutan Cortex. Paeoniflorin, recognized as the principal active compound, originates predominantly from Paeoniae Radix Alba. Benzoic acid is present in multiple herbal components, including Cinnamomi Ramulus and Poria.

**TABLE 2:** Analysis results of 10 main components in Guizhi Fuling Pills.

ID	NameEN	Formula	mzmed	rtmed	ppm	Type	MS2
1	Paeonol	C9H10O3	149.0593	97.2	3	POS	103.1; 107; 131
2	Amygdalin	C20H27NO11	456.1508	239.9	0.7	NEG	89; 59; 101
3	Catechin	C15H14O6	289.0717	241.2	0.2	NEG	289.1; 245.1; 125
4	Paeonolide	C20H28O12	459.1509	257.2	0.2	NEG	161.1; 456.1; 125
5	Paeoniflorin	C23H28O11	479.1554	268.6	0.9	NEG	121; 165.1; 327.1
6	Propylgallate	C10H12O5	211.0613	305	0.4	NEG	153.1; 152; 211.1
7	Benzoic acid	C7H6O2	121.0295	309.6	0.2	NEG	121; 106
8	Mudanpioside C	C30H32O13	599.177	324.6	0.1	NEG	137; 281.1; 121
9	Benzoylpaeoniflorin	C30H32O12	583.1822	348.3	0.2	NEG	121; 165.1
10	3-Phenylpropanoic acid	C9H10O2	149.0608	352.8	0.1	NEG	149.1; 134; 107.1

### 3.2 Guizhi Fuling Pills reduced DDP-induced renal injury

**FIGURE 2:**
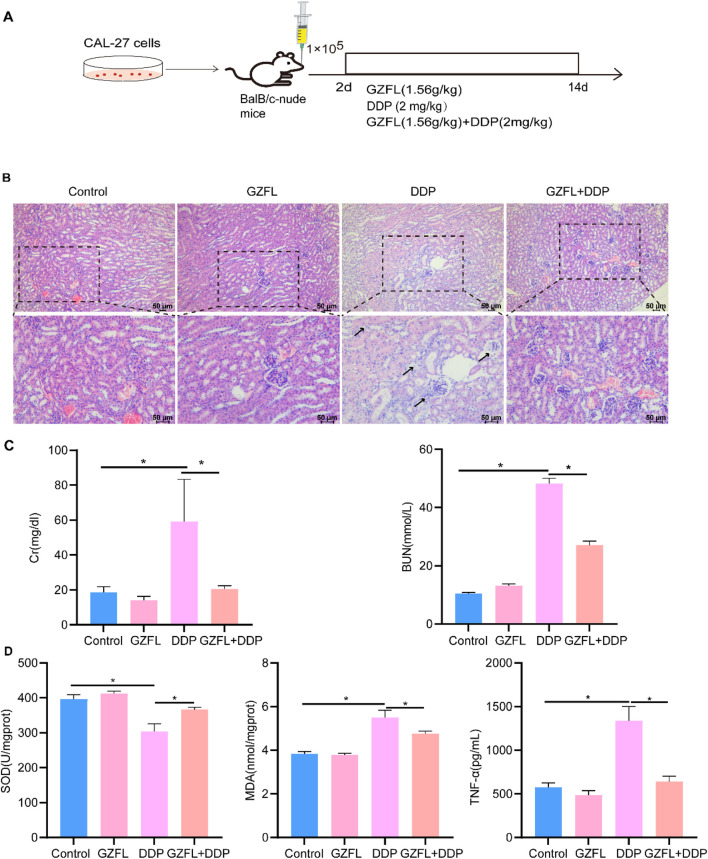
Guizhi Fuling Pills reduced DDP-induced renal injury. **(A)** Modeling and intervention process of BalB/c-nude mice. The mice were divided into four groups (n = 5/group): model group (control), Guizhi Fuling Pills treatment group (GZFL), DDP treatment group (DDP), and Guizhi Fuling Pills combined with DDP treatment group (GZFL + DDP). **(B)** HE staining results of kidneys in each group (scale: 50 µm). The arrow indicates inflammatory infiltration and vacuolar lesions of renal tissue. **(C)** The changes in creatinine (CRE) and blood urea nitrogen (BUN) levels in mouse serum showed that the DDP group had significantly higher creatinine levels than the other groups. **(D)** The level of superoxide dismutase (SOD) in the DDP group was significantly lower than that in the control group, while the level of SOD in the Guizhi Fuling pill combined with the DDP group was higher than that in the DDP group. The levels of MDA and TNF-α in the DDP group were significantly higher than those in the control group, while the levels of malondialdehyde (MDA) and tumor necrosis factor-alpha (TNF-α) in Guizhi Fuling Pill combined with the DDP group were lower than those in the DDP group (n = 4). One-way analysis of variance (one-way ANOVA) was used to analyze the means between groups. All data are presented as mean ± SD. Statistical significance is indicated as follows: *P < 0.05. GZFL, Guizhi Fuling Pills.

Cisplatin induces nephrotoxicity, leading to irreversible renal injury (Jing et al., 2021). To evaluate the potential protective effects of Guizhi Fuling Pills against DDP-induced nephrotoxicity, Cal27 cells (2 × 10^5^) were injected into the submucosal layer of the mid-tongue region in BALB/c nude mice. Subsequently, the mice were administered GZFL, DDP, or a combination of Guizhi Fuling Pills and cisplatin (denoted as GZFL + DDP) (Figure 2A). DDP treatment induced prominent inflammatory infiltration and vacuolar lesions in renal histopathological sections compared with the control group (Chenxu et al., 2018) (Figure 2B). Meanwhile, the serum levels of CRE and BUN in the DDP group were significantly higher than those in the control group (Figure 2C). Renal tissues from the DDP group showed a significant reduction in the SOD activity, accompanied by elevated levels of MDA and TNF-α, relative to those of the control group (Figure 2D). These results demonstrate that DDP induced significant renal injury, while coadministration of Guizhi Fuling Pills with DDP markedly attenuated renal inflammatory infiltration and vacuolar degeneration (Figure 2B). The serum CRE and BUN levels in the GZFL + DDP group were significantly reduced compared to those in the DDP group (CRE *P* = 1.36 × 10^−3^, *P* < 0.05; BUN *P* = 6.80 × 10^−11^, *P* < 0.05) (Figure 2C). The level of SOD was increased (*P* = 1.00 × 10^−4^, *P* < 0.05), while the levels of MDA and TNF-α were decreased in the GZFL + DDP group compared to the DDP group (MDA: *P* = 7.65 × 10^−4^, *P* < 0.05; TNF-α: *P* = 1.01 × 10^−6^, *P* < 0.05) (Figure 2D). In summary, Guizhi Fuling Pills ameliorated DDP-induced renal injury through antioxidant and anti-inflammatory mechanisms.

### 3.3 Guizhi Fuling Pills improved the inhibitory effect of cisplatin on TSCC

**FIGURE 3:**
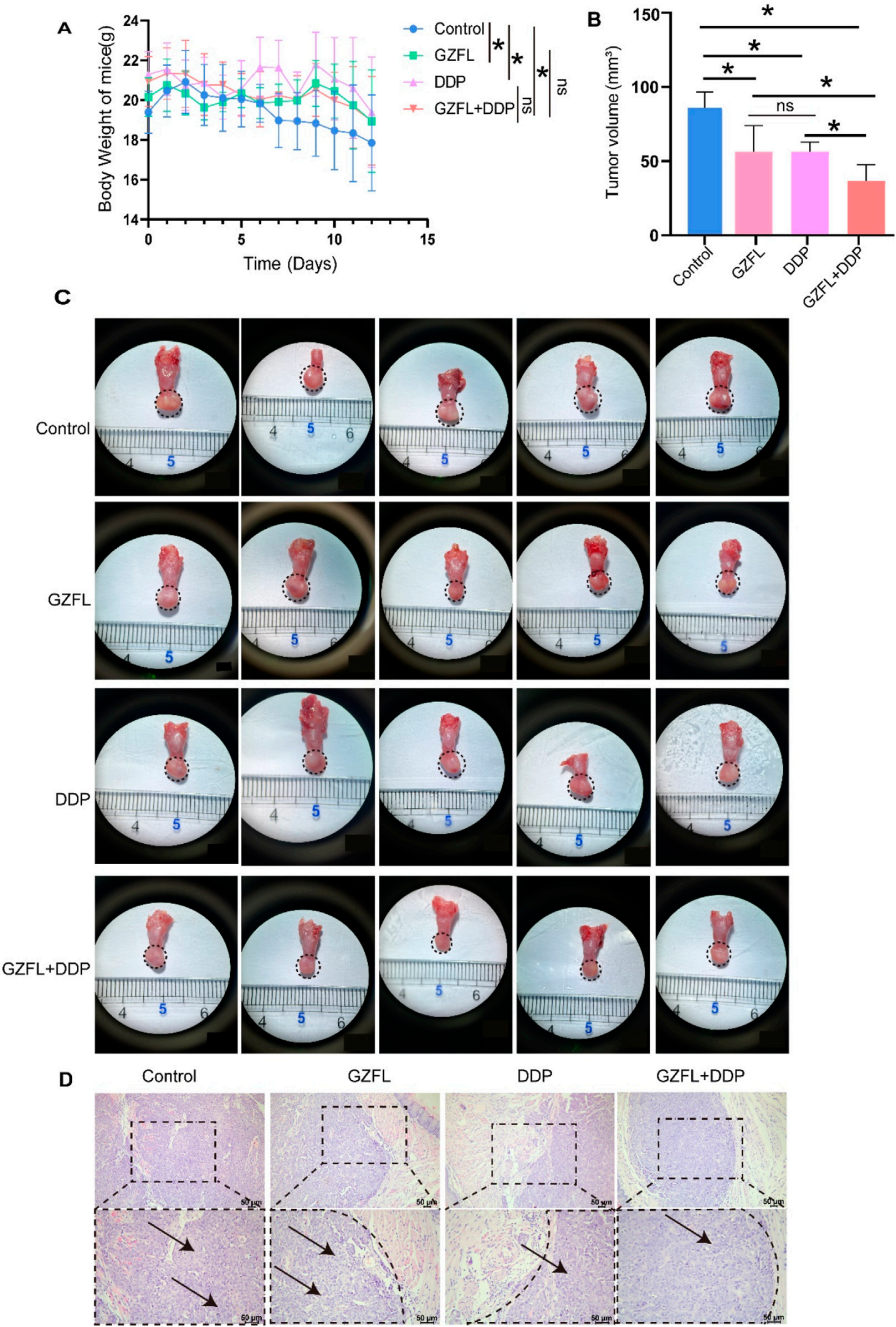
Guizhi Fuling Pills improve the inhibitory effect of cisplatin on TSCC. **(A)** The weight changes of TSCC mice treated with Guizhi Fuling Pills, DDP, and Guizhi Fuling Pills combined with DDP over time were observed. **(B)** The histogram was used to summarize the tongue tumor volume after treatment with Guizhi Fuling Pills and DDP. The tumor volume in the treatment group was significantly lesser than that in the control group. **(C)** Cal-27 cells were injected into the tongue of immunodeficient mice. After the intervention of Guizhi Fuling Pills and DDP, the tongue tissue of mice was dissected (the area around the virtual coil represents the tumor tissue) (n = 5). **(D)** HE staining can be used to observe tumor lesions in each group. The results showed that the tumor cell volume increased and the cytoplasm was loose, forming balloon-like changes. The area around the virtual coil represents the tumor tissue. The arrows mark the tumor cells with obvious characteristics. One-way analysis of variance (one-way ANOVA) was used to analyze the means between groups. All data are presented as mean ± SD. Statistical significance is indicated as follows: ns, not significant; *P < 0.05. GZFL, Guizhi Fuling Pills.

The body weight of nude mice significantly increased in the GZFL (20.38 g ± 0.56 g) (*P* = 2.18 × 10^−2^), DDP (20.75 g ± 0.86 g) (*P* = 5.13 × 10^−4^), and GZFL + DDP groups (20.22 g ± 0.86 g) (*P* = 2.71 × 10^−2^) compared to the control group (19.49 g ± 0.56 g) (*P* < 0.05). This result indicates that Guizhi Fuling Pills, alone or in combination with DDP, increased body weight in tumor-bearing nude mice (Figure 3A). Furthermore, tumor volume was significantly reduced in the GZFL (*P* = 2.12 × 10^−4^, *P* < 0.05), DDP (*P* = 1.08 × 10^−4^, *P* < 0.05), and GZFL + DDP groups (*P* = 6.00 × 10^−8^, *P* < 0.05) compared with the control group (Figures 3B,C). Notably, no significant difference in tumor volume was observed between the GZFL (50.85 mm^3^ ± 15.92 mm^3^) and DDP (50.84 mm^3^ ± 5.907 mm^3^) groups (*P* = 8.13 × 10^−1^, *P* > 0.05) (Figure 3B). These results demonstrate that Guizhi Fuling Pills exhibited antitumor efficacy comparable to that of DDP in suppressing tumor growth. Moreover, the combined treatment of GZFL and DDP resulted in a 38% reduction in tumor volume (32.80 ± 10.07 mm^3^) compared to the control group, 0.35 times that of the GZFL group, and 0.35 times that of the DDP group (Figure 3B). Histopathological analysis of HE-stained sections revealed tumor areas, consistent with the preceding results (Figure 3D). Combination therapy with Guizhi Fuling Pills and DDP significantly suppressed tumor growth *in vivo*.

### 3.4 Guizhi Fuling Pills regulated intestinal flora

**FIGURE 4:**
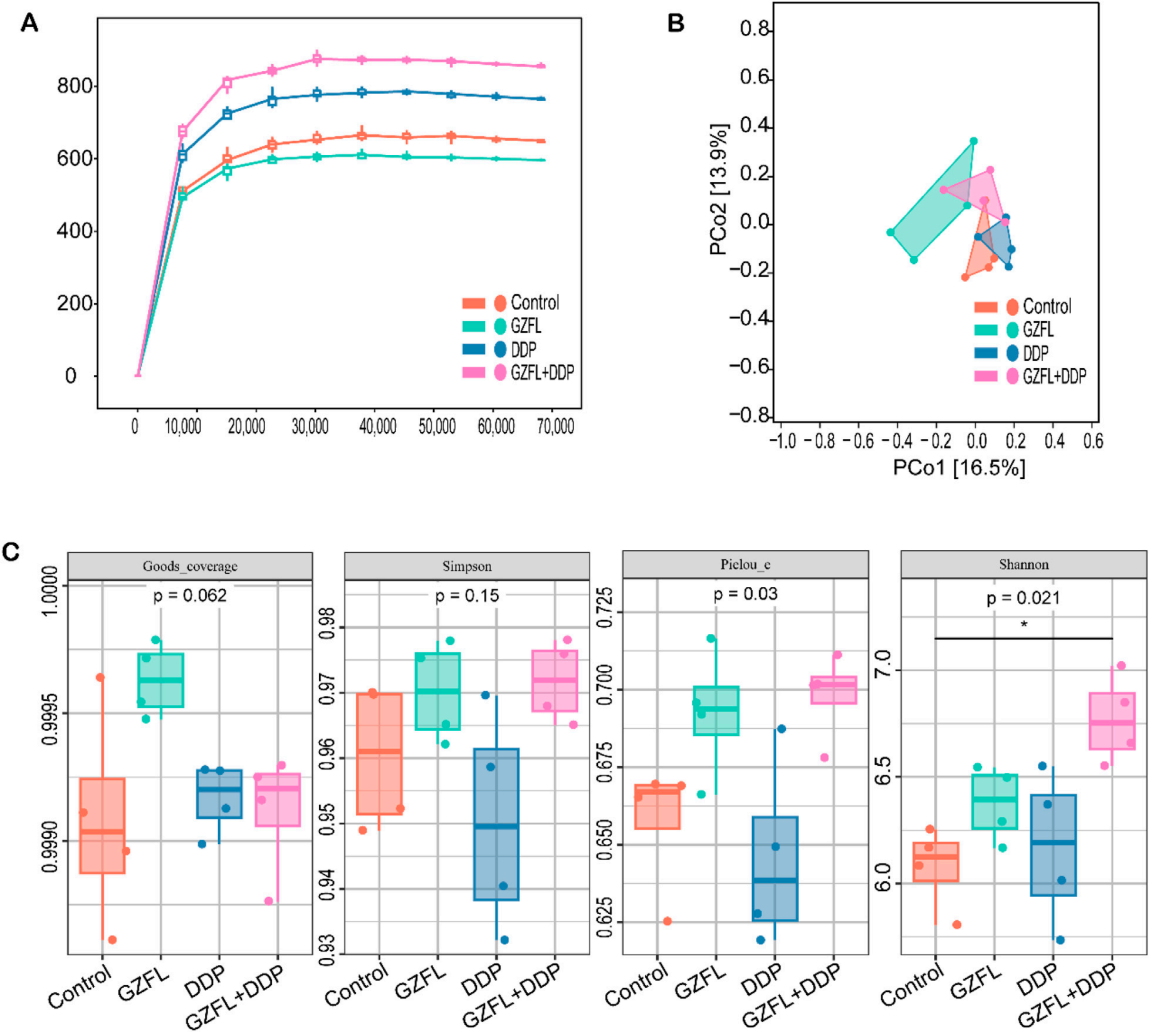
Guizhi Fuling Pills regulated the gut microbiota of mice. **(A)** The sparse curve is close to the plateau period, indicating that the sequencing depth is sufficient to cover almost all the microbial species present in the sample. This indicates that the sequencing data are reliable (n = 4). **(B)** Through principal coordinate analysis (PCoA), the GZFL group has better dispersion than the control, DDP, and GZFL + DDP groups (n = 4). **(C)** Alpha diversity index (Goods-coverage, Simpson, Piclou-e, and Shannon) of different groups of mice (n = 4). Statistical significance is indicated as follows: ns, not significant; *P < 0.05. GZFL, Guizhi Fuling Pills.

Accumulating evidence suggests that gut microbiota dysbiosis plays a role in the pathogenesis of kidney injury (Zhou et al., 2024). In this study, 16S rRNA sequencing of mouse fecal samples was performed. The rarefaction curve reached saturation, demonstrating that the sequencing depth adequately covered the majority of microbial species in the sample (Figure 4A). The sequencing data are, therefore, reliable. The DDP and GZFL + DDP groups showed higher species richness than the control group (Figure 4A). However, the GZFL group showed lower species richness than the control group. The species richness of GZFL + DDP was higher than that of both the DDP and GZFL groups. The results showed that Guizhi Fuling Pills combined with DDP increased the species richness of mice. By principal coordinate analysis (PCoA), the dispersion of the GZFL group was better than that of the control, DDP, and GZFL + DDP groups (Figure 4B). The results indicated that the microbial community of the GZFL group was markedly different from that of other groups. The DDP and GZFL + DDP groups were more dispersed than the control group. These results further demonstrate that treatment with DDP alone or in combination with GZFL altered the gut microbial community.

The Goods_coverage index reflects the sequencing depth. There was no significant difference in the Goods_coverage index in each group, indicating that there was no significant difference in the depth of sequencing in each group (*P* = 6.20 × 10^−2^, *P* > 0.05). The Simpson index is one of the indices used to estimate the microbial diversity in samples. There was no significant difference in Simpson index among the four groups, indicating that there was no significant difference in microbial diversity in the samples (*P* = 1.50 × 10^−1^, *P* > 0.05). The Pielou_e index is used to measure species evenness. There was no significant difference in the Pielou_e index between the GZFL , DDP, and GZFL + DDP groups compared with that in the control group. At the same time, the Pielou_e index of the GZFL group was not different from that of the GZFL + DDP group. However, the Pielou_e index of the GZFL + DDP group was significantly higher than that of the DDP group (*P =* 3.00 × 10^−2^, *P >* 0.05). These results indicate that the combination of Guizhi Fuling Pills and DDP altered the evenness of intestinal flora after DDP treatment.

The Shannon index, which reflects both microbial richness and evenness, showed no significant differences in the DDP or GZFL groups when compared to the control group (Figure 4C). There was no significant difference in the Shannon index between the GZFL group and the GZFL + DDP group. However, the Shannon index of the GZFL + DDP group was higher than that of the control group (*P =* 2.10 × 10^−2^, *P >* 0.05). These findings suggest that concurrent administration of Guizhi Fuling Pills with DDP enhances intestinal bacterial diversity during DDP treatment.

### 3.5 Guizhi Fuling Pills promoted gut microbiota homeostasis

**FIGURE 5:**
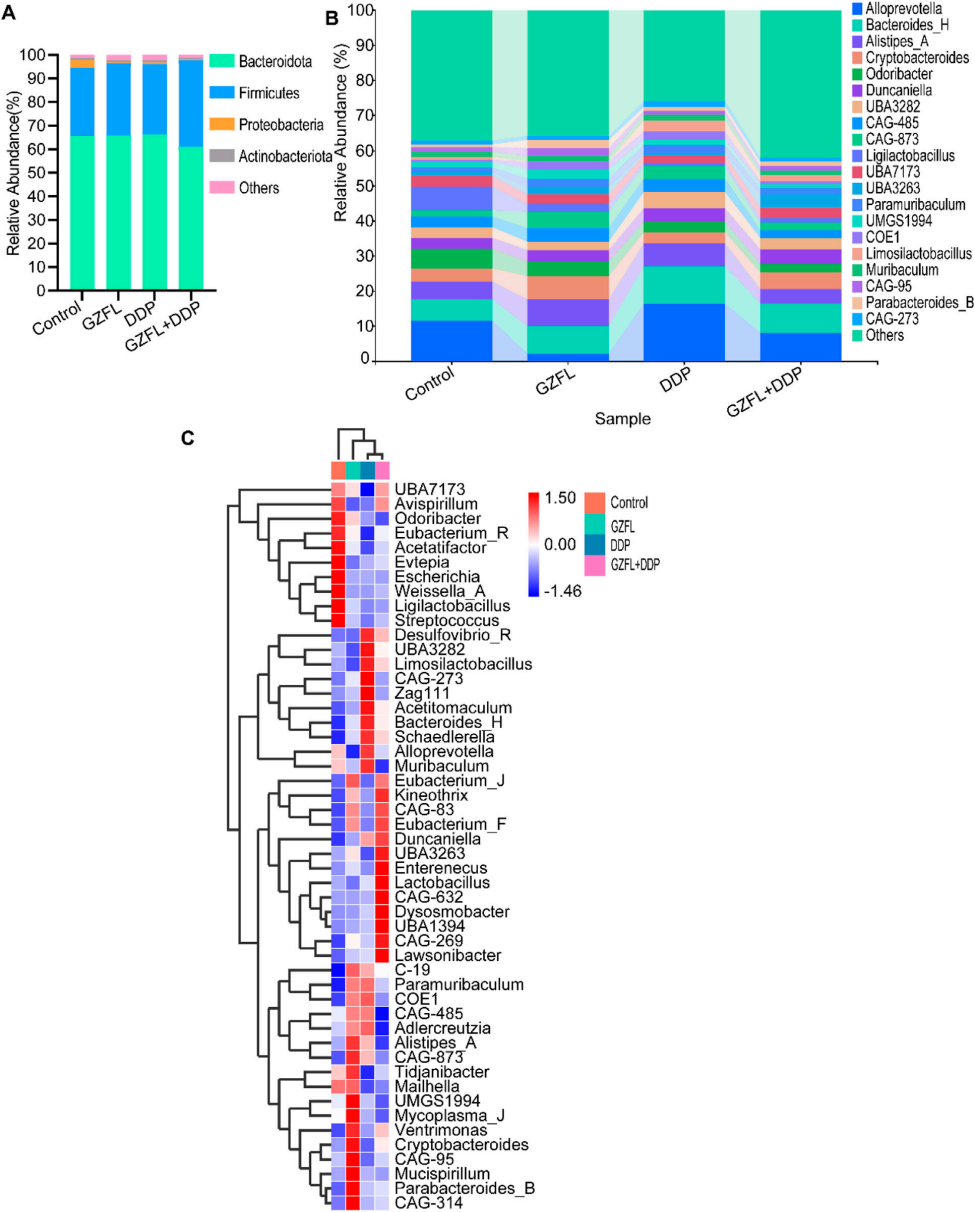
Guizhi Fuling Pills regulated gut microbiota homeostasis. **(A)** At the phylum level of species taxonomy, changes in the F/B ratio of the combined group indicate alterations in its microbial community structure. **(B)** Distribution of the species taxonomic composition at the genus level. **(C)** Heatmaps of the top 50 species in each group of mice are displayed in terms of species composition. Data are shown as mean value (n = 4 per group). GZFL, Guizhi Fuling Pills.

The ratio of *Firmicutes*/*Bacteroidetes* (F/B) reflected gut flora structure (Mariat et al., 2009). The F/B ratio was significantly higher in the GZFL + DDP group than in the control, GZFL, and DDP groups (Figure 5A). Interestingly, the proportion of *Proteobacteria* was higher in the control group than in the GZFL, DDP, and GZFL + DDP groups (Figure 5A). These results show that the combination of Guizhi Fuling Pills and DDP optimized gut flora structure.

The abundance of *Alloprevotella* was higher in cancer tissues and has been implicated as a factor associated with oral squamous cell carcinoma (Zhang et al., 2019a). At the genus level, the proportion of *Alloprevotella* was different among the four groups (Figure 5B). The abundance of *Alloprevotella* was significantly lower in the GZFL group compared to the control, DDP, and GZFL + DDP groups. Meanwhile, the abundance of *Alloprevotella* was reduced in the GZFL + DDP group compared with the DDP group (Figure 5B). These findings suggest that Guizhi Fuling Pills reduced the abundance of *Alloprevotella* during DDP treatment.

The abundance of *Desulfovibrio*, a harmful bacterium associated with acute intestinal injury (Han et al., 2021), was also increased in the DDP group compared with the control and GZFL groups. In contrast, the abundance of *Desulfovibrio* decreased in the GZFL + DDP group compared with the DDP group, suggesting that Guizhi Fuling Pills reduced the abundance of *Desulfovibrio* caused by DDP.

*Parabacterioids B* (Chang et al., 2019) is recognized as a genus with promising probiotic activity, demonstrating protective roles in several diseases (Cekanaviciute et al., 2017), colorectal cancer (Koh et al., 2020), and inflammatory bowel disease (Kverka et al., 2011). The abundance of *Parabacterioids B* in the GZFL group was increased compared with that in the control, DDP and GZFL + DDP groups (Figure 5C).

In addition, the abundance of several probiotic genera, including *Lactobacillus*, *Kineothrix*, and *Eubacteria* (Liu X. et al., 2024; Huang et al., 2022; Gou et al., 2023), was upregulated in the GZFL + DDP group. *Lactobacillus* confers health benefits in the gastrointestinal tract through multiple mechanisms, such as inhibiting pathogens, sustaining microbial equilibrium, modulating immune responses, and strengthening the intestinal epithelial barrier (Sengupta et al., 2013). Moreover, previous studies have reported that *Lactobacillus* and *Kineothrix* contribute to increased production of SCFAs and amelioration of renal injury. The abundance of *Lactobacillus* was higher in the GZFL + DDP group than in the control, GZFL, and DDP groups. These results suggest that Guizhi Fuling Pills increased the abundance of *Lactobacillus*, *Eubacteria*, *Kineothrix*, and *Parabacteroides B* during DDP treatment.

### 3.6 Guizhi Fuling Pills improved intestinal inflammation caused by DDP

**FIGURE 6:**
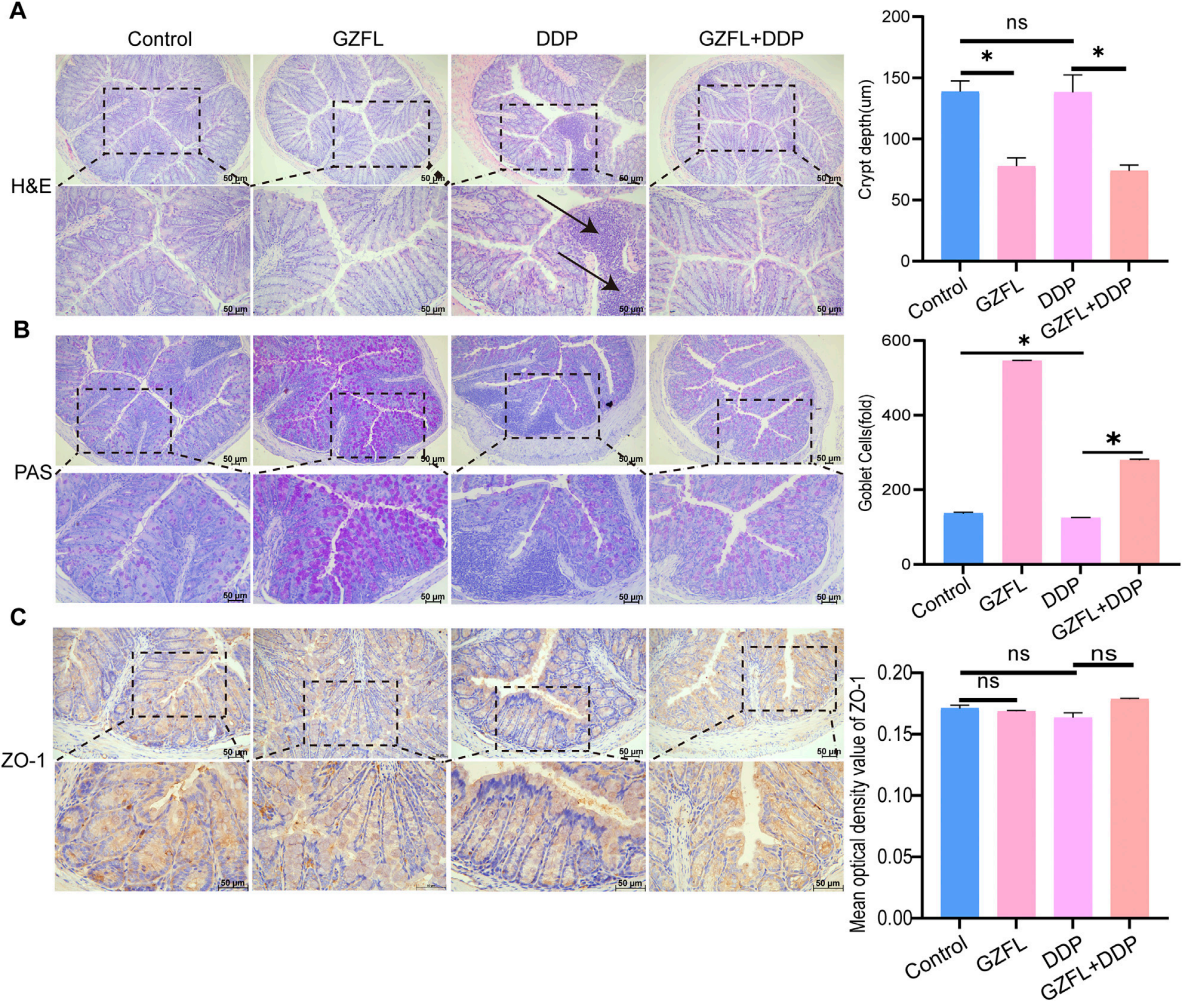
Guizhi Fuling Pills improved intestinal inflammation caused by DDP **(A)** HE staining observation of colonic histopathology and depth of the crypt in the colon (n = 3); the DDP group showed obvious inflammatory infiltration (position marked by black arrows). **(B)** Observation of the number of goblet cells in the colon of mice using PAS staining (n = 3). **(C)** Observation of mouse colon tight junction protein using ZO-1 staining (scale: 50 µm) (n = 3). One-way analysis of variance was used to analyze the means between groups. All data are presented as mean ± SD. Statistical significance is indicated as follows: ns, not significant; *P < 0.05. GZFL, Guizhi Fuling Pills.

To evaluate intestinal inflammation related to gut microbiota changes, colon tissues were analyzed using HE staining. DDP treatment resulted in marked inflammatory infiltration relative to both the control and GZFL groups, demonstrating its proinflammatory action in the gut. Guizhi Fuling Pills did not induce inflammatory infiltration in the gut. However, the GZFL + DDP group had obvious inflammatory infiltration compared with the GZFL group. The extent of inflammatory infiltration in the GZFL + DDP group was reduced compared with that in the DDP group. GZFL + DDP treatment reduced the depth of intestinal crypts compared with the DDP group, and of the shallow crypts, suggesting that both the cell maturation rate and the secretion function increased (*P* = 7.64 × 10^−4^, *P* < 0.05). Collectively, these results demonstrate that Guizhi Fuling Pills ameliorated DDP-induced intestinal inflammation (Figure 6A).

Gut goblet cells separate the intestinal epithelium from commensal bacteria and contribute to maintaining microbiota homeostasis and immune regulation (Birchenough et al., 2015). To observe the number of goblet cells in different treatment groups, we performed PAS staining. The number of goblet cells in the DDP group was lower than that in the control group (*P* = 2.30 × 10^−3^, *P* < 0.05) (Figure 6B), while goblet cell numbers were higher in the GZFL + DDP group than in DDP alone (*P* = 1.21 × 10^−13^, *P* < 0.05) (Figure 6B). These results indicate that Guizhi Fuling Pills enhanced intestinal mucosal secretion function during DDP treatment. ZO-1 is a key structural protein of tight junctions (Kuo et al., 2022). Reduced expression or impaired function of ZO-1 compromises tight junction integrity and weakens gut barrier defense (Kuo et al., 2022). There was no significant difference in the expression level of ZO-1 between the GZFL (*P* = 2.28 × 10^−1^, *P* > 0.05), DDP (*P* = 9.96 × 10^−2^, *P* > 0.05), and GZFL + DDP groups (*P* = 6.51 × 10^−2^, *P* > 0.05) compared with that in the control group (Figure 6C). These results showed that Guizhi Fuling Pills and DDP do not affect the intestinal barrier.

## 4 Discussion

TSCC is among the most aggressive oral cancers, accounting for approximately 41% of all oral malignant tumors. DDP is a first-line chemotherapeutic agent for TSCC, but its use is limited by substantial nephrotoxicity (Jing et al., 2021). This study demonstrates that Guizhi Fuling Pills attenuate DDP-induced renal injury and enhance its antitumor efficacy against TSCC. These beneficial effects are associated with modulation of gut microbiota, reduction of inflammation, and improvement of intestinal mucosal secretion.

DDP induces multi-organ damage, notably nephrotoxicity and intestinal injury. Its renal toxicity is well established in cancers such as cervical and lung cancers (Huo et al., 2022; Zhang et al., 2019b). We also found that DDP caused renal damage during the treatment in nude mice with TSCC. Previous studies have demonstrated that Guizhi Fuling Pills significantly alleviate chemical-induced nephrotoxicity, including models of CCl_4_ exposure (Xu et al., 2022). Our study demonstrated that Guizhi Fuling Pills significantly alleviated renal injury induced by DDP, as evidenced by reduced inflammatory infiltration and vacuolar degeneration. After treatment with Guizhi Fuling Pills, the serum levels of creatinine and urea nitrogen decreased, accompanied by increased SOD activity, as well as decreased MDA and TNF-α levels. Guizhi Fuling Pills mitigated DDP-induced renal injury via anti-inflammatory and antioxidant mechanisms. Furthermore, Guizhi Fuling Pills significantly reduced orthotopic tongue tumor volume in nude mice. The combination of Guizhi Fuling Pills with DDP resulted in superior tumor growth suppression. Similarly, Guizhi Fuling Pills inhibited tumor growth in bladder cancer (Lu et al., 2016), cervical cancer (Yao and Shulan, 2008), breast cancer (Dai et al., 2020), and ovarian cancer (Ma et al., 2024). Furthermore, Guizhi Fuling Pills also suppressed tumor growth in patients with ovarian cancer (Liu W. et al., 2024) and uterine fibroids (Meng et al., 2022). Emerging evidence indicates an association between renal injury and gut microbiota (Zhou et al., 2024; Kobayashi et al., 2021; Wang et al., 2023). At the same time, previous studies have demonstrated that certain drugs alleviate DDP-induced nephrotoxicity via the gut microbiota (Tian et al., 2024). The main metabolites of Guizhi Fuling Pills include paeonol, amygdalin, paeoniflorin, cinnamic acid, catechin, and benzoic acid (Chen et al., 2009). Studies have shown that paeoniflorin and cinnamaldehyde decrease the abundance of Desulfovibrio in the gut microbiota (Liu et al., 2020; Gao et al., 2022; Zhang et al., 2024). DDP increased Desulfovibrio abundance, whereas Guizhi Fuling Pills reduced it. The Guizhi Fuling Pills group exhibited reduced species richness, with no significant alteration in the flora structure compared to the control group. Consistent with previous reports that catechin in Guizhi Fuling Pills increases *Parabacteroides* abundance (Luo et al., 2018), we observed elevated *Parabacteroides-B* levels following treatment. Gallic acid (Kim et al., 2021) and paeonol (Zheng et al., 2022) increased the abundance of *Lactobacillus* and exerted anti-inflammatory effects.

Combination treatment with Guizhi Fuling Pills and DDP increased species richness, improved flora structure, and elevated *Lactobacillus* abundance. The combination of Guizhi Fuling Pills and DDP elevated abundances of *Kineothrix* and *Lactobacillus*, enhanced SCFA production, and attenuated renal injury (Liu X. et al., 2024). Notably, the combination of Guizhi Fuling Pills and DDP enhanced species evenness and microbial diversity and reduced the abundance of the harmful bacterium Desulfovibrio.

Given the reported detrimental effects of Desulfovibrio on intestinal integrity, we performed histological staining to assess intestinal damage. DDP treatment induced severe inflammatory infiltration in the intestinal tissue. This observation aligns with previous reports that DDP reduces small intestinal goblet cells and glands (Tian et al., 2024). Guizhi Fuling Pills reduced the intestinal inflammatory infiltration caused by DDP. DDP reduced the number of goblet cells, while Guizhi Fuling Pills combined with DDP increased the number of goblet cells. No significant differences in ZO-1 expression were observed among the groups. In summary, our study demonstrates that DDP treatment reduced bacterial richness, disrupted flora structure, and diminished goblet cell numbers, resulting in decreased mucus secretion. Guizhi Fuling Pills mainly improved the structure of intestinal flora and increased the secretion of intestinal mucus. We must admit a key limitation of this study. We must acknowledge several limitations in this study. To explore the improvement of Guizhi Fuling Pills on DDP-induced renal injury, we chose the commonly used dose for humans to convert it into the dose of mice for experiments. Our study demonstrated that the combination of Guizhi Fuling Pills and DDP ameliorated DDP-induced renal injury, correlating with changes in the abundance of *Kineothrix*, *Lactobacillus*, and *Desulfovibrio*. However, the specific contributions of individual bacterial species require further clarification. Future work will use antibiotic-treated mouse models to establish causal links between microbial shifts and therapeutic effects. Additionally, drug stability remains to be fully characterized; subsequent studies will include systematic HPLC-based stability assessments. This study showed that Guizhi Fuling Pills combined with DDP attenuated renal injury and potentiated its antitumor efficacy in TSCC. Therefore, the combination of Guizhi Fuling Pills and DDP represents a promising choice for the treatment of TSCC.

## Data Availability

The raw data supporting the conclusions of this article will be made available by the authors, without undue reservation.
