# Supplementary material for: Traditional Chinese medicine prescription Guizhi Fuling Pills ameliorate cisplatin-induced renal injury via remodeling intestinal homeostasis in mice with tongue squamous cell carcinoma
Source: Front Pharmacol. 2025 Oct 2;16:1631966. doi: 10.3389/fphar.2025.1631966 (PMC12528071; doi:10.3389/fphar.2025.1631966)
Supplement: Supplementary file 1 [file Supplementaryfile1.docx]

MS2 Spectra of Main Components in Guizhi Fuling Pills

Figure S1


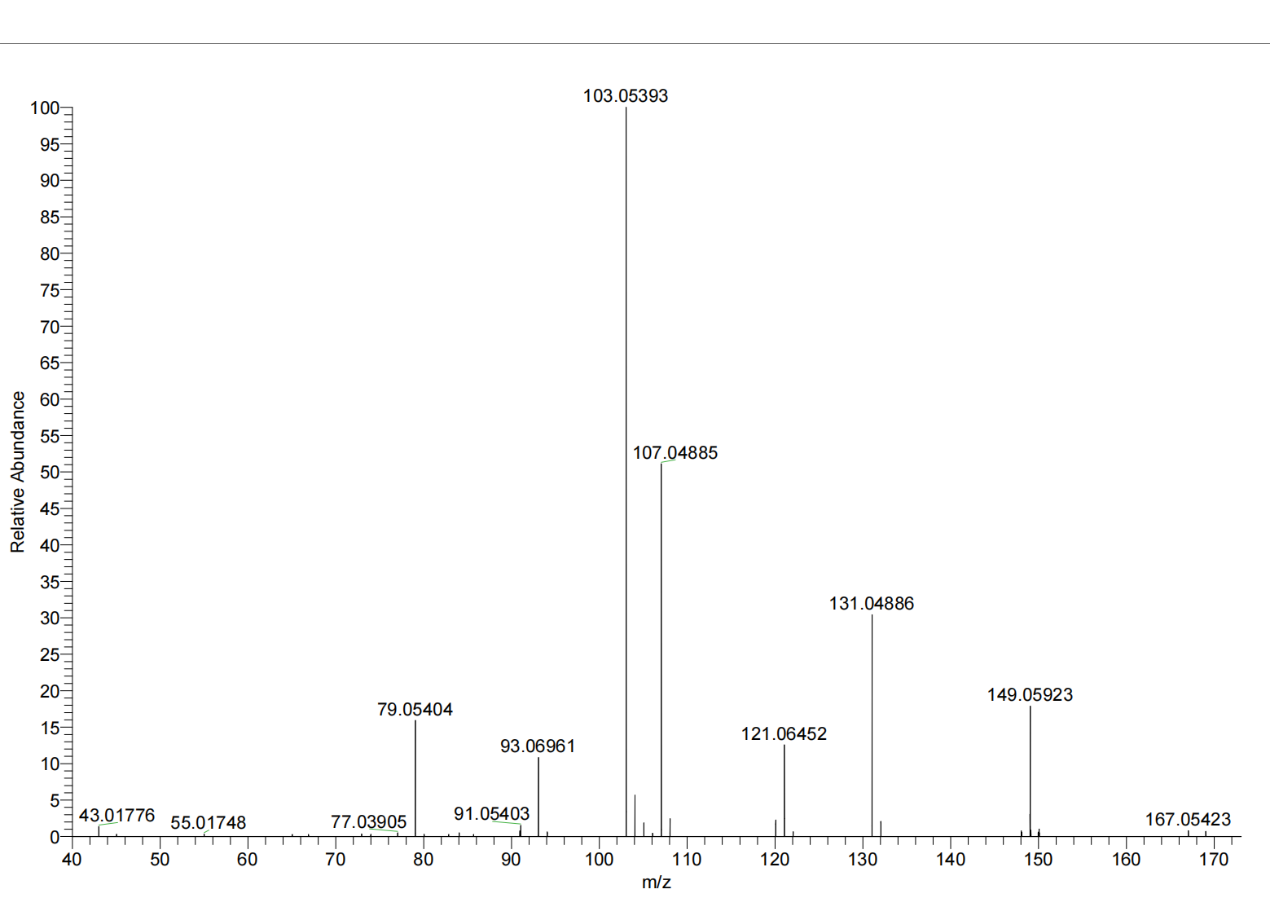


Fig.S1 MS2 Spectrum of Paeonol.Compound Information: Paeonol (C₉H₁₀O₃), a characteristic component of Moutan Cortex in Guizhi Fuling Pills. Mass Spectrometry Parameters: Secondary mass spectrum (MS2) acquired in positive ionization mode (POS) (consistent with Table 2 in the main text).Axis Definitions:X-axis: Mass-to-charge ratio (m/z), representing the mass of fragment ions relative to their charge. Y-axis: Relative Abundance (%), indicating the relative content of each fragment ion (normalized to the most abundant ion as 100%).Characteristic Fragment Ions: Key peaks correspond to m/z 149.05923 (molecular ion peak), 131.04886, 107.04885, 103.05393, etc.

Figure S2


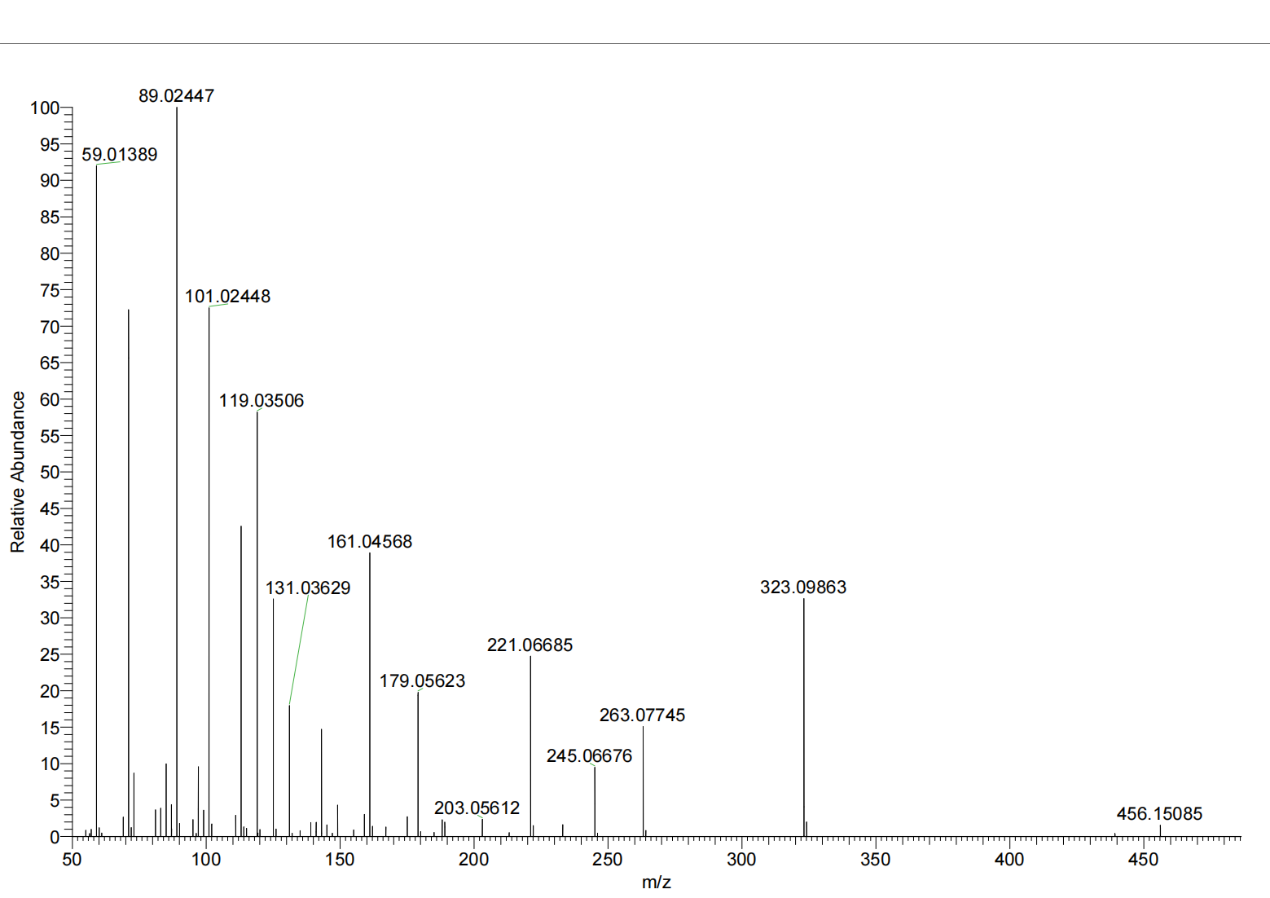


Fig. S2 MS2 Spectrum of Amygdalin.

Compound Information: Amygdalin (C₂₀H₂₇NO₁₁), a marker component derived from Persicae Semen in Guizhi Fuling Pills. Mass Spectrometry Parameters: Secondary mass spectrum (MS2) acquired in negative ionization mode (NEG) (consistent with Table 2 in the main text).Axis Definitions: X-axis: Mass-to-charge ratio (m/z), representing the mass of fragment ions relative to their charge. Y-axis: Relative Abundance (%), indicating the relative content of each fragment ion (normalized to the most abundant ion as 100%).Characteristic Fragment Ions: Key peaks correspond to m/z 456.15085 (molecular ion peak), 101.02448, 89.02447, 59.01389, etc., matching the standard MS2 profile of amygdalin.

Figure S3


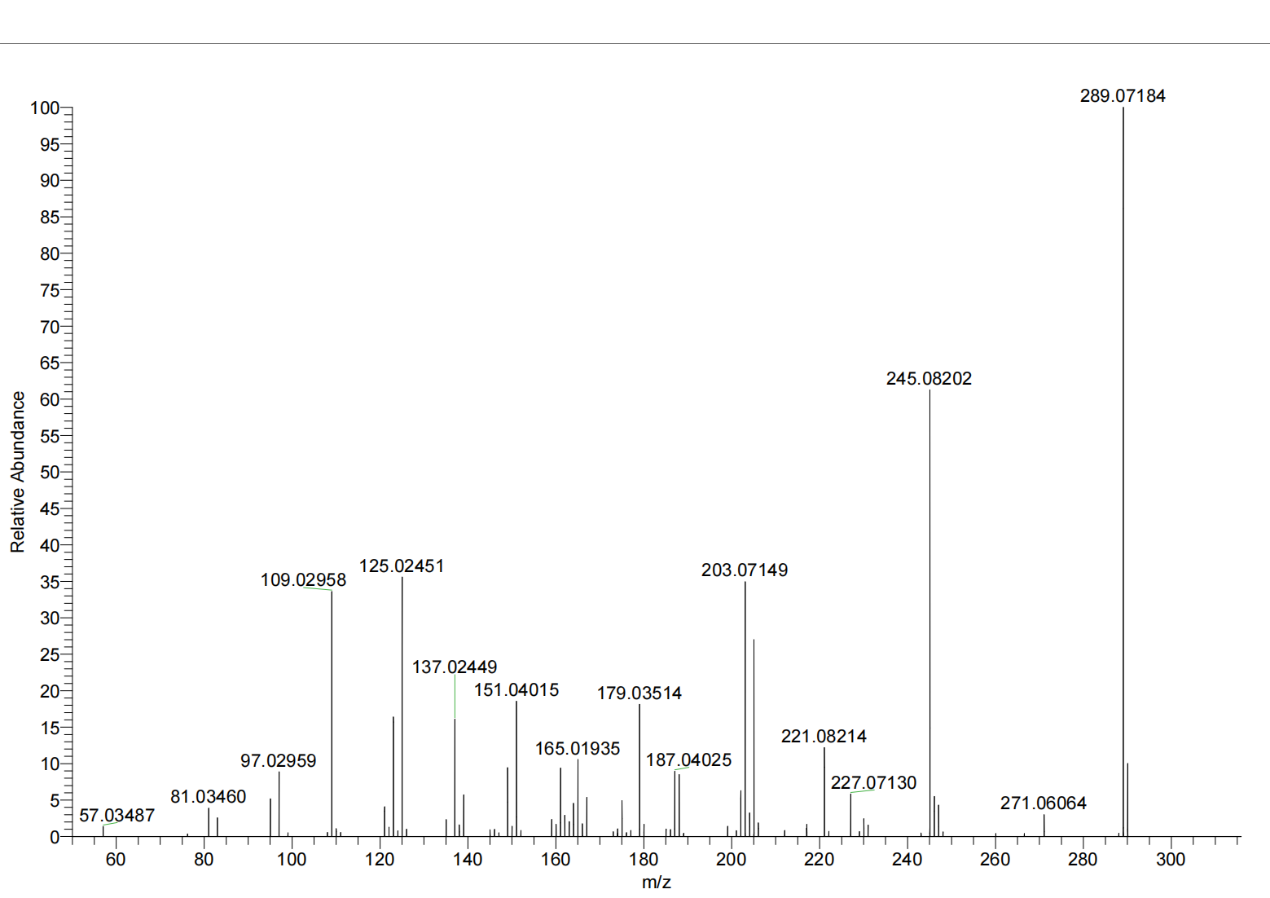


Fig. S3 MS2 Spectrum of Catechin Compound Information: Catechin (C₁₅H₁₄O₆), a common component in Paeoniae Radix Alba and Moutan Cortex of Guizhi Fuling Pills. Mass Spectrometry Parameters: Secondary mass spectrum (MS2) acquired in negative ionization mode (NEG) (consistent with Table 2 in the main text). Axis Definitions: X-axis: Mass-to-charge ratio (m/z), representing the mass of fragment ions relative to their charge. Y-axis: Relative Abundance (%), indicating the relative content of each fragment ion (normalized to the most abundant ion as 100%). Characteristic Fragment Ions: Key peaks correspond to m/z 289.07184 (molecular ion peak), 245.08202, 125.02451, etc., which are typical fragmentation features of catechin.

Figure S4


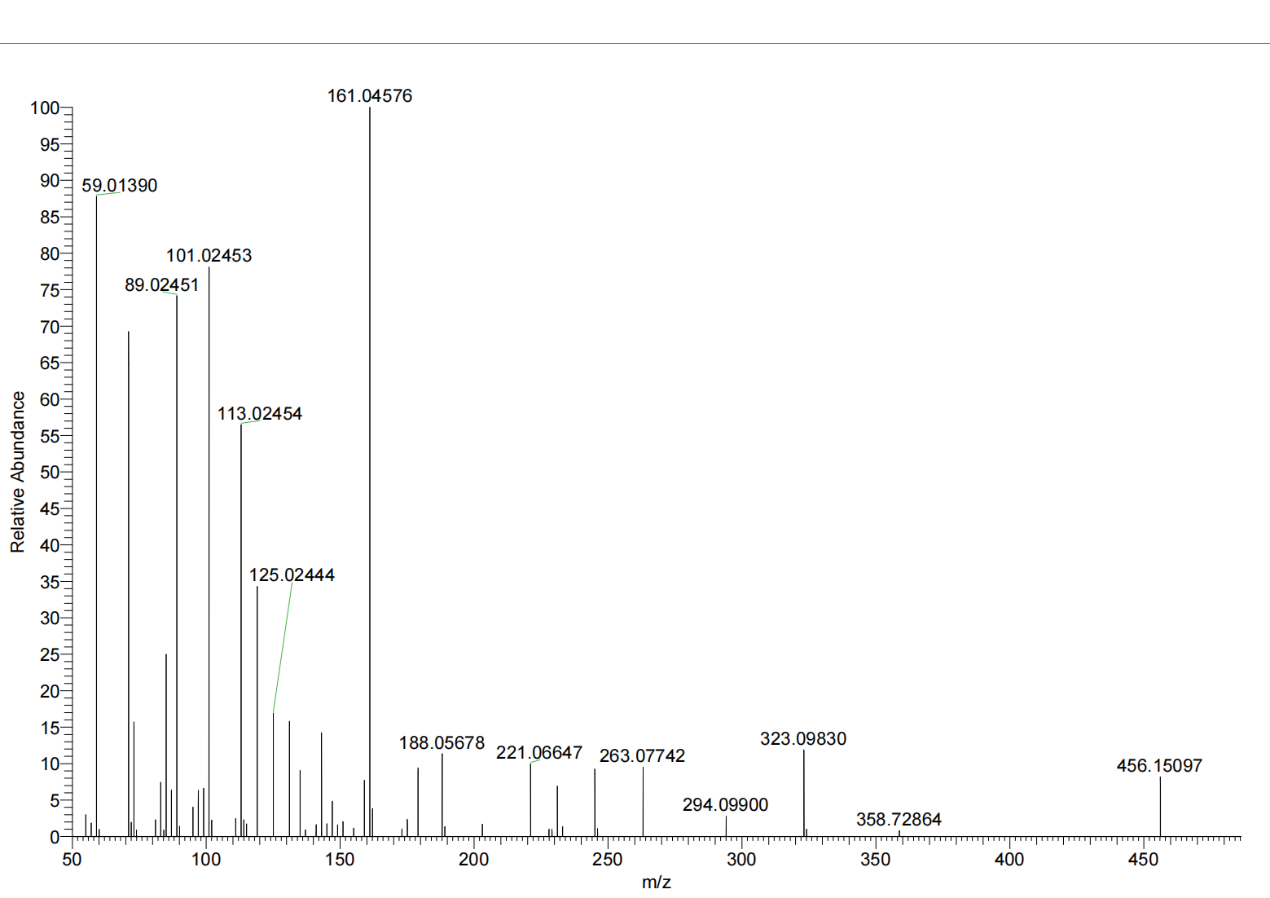


Fig. S4 MS2 Spectrum of Paeonolide. Compound Information: Paeonolide (C₂₀H₂₈O₁₂), an active component of Moutan Cortex in Guizhi Fuling Pills. Mass Spectrometry Parameters: Secondary mass spectrum (MS2) acquired in negative ionization mode (NEG) (consistent with Table 2 in the main text). Axis Definitions: X-axis: Mass-to-charge ratio (m/z), representing the mass of fragment ions relative to their charge. Y-axis: Relative Abundance (%), indicating the relative content of each fragment ion (normalized to the most abundant ion as 100%). Characteristic Fragment Ions: Key peaks correspond to m/z 456.15097 (molecular ion peak), 161.04576, 125.02444, etc. (consistent with the MS2 information in Table 2 of the main text), reflecting the glycoside cleavage and benzene ring fragmentation of paeonolide.

Figure S5


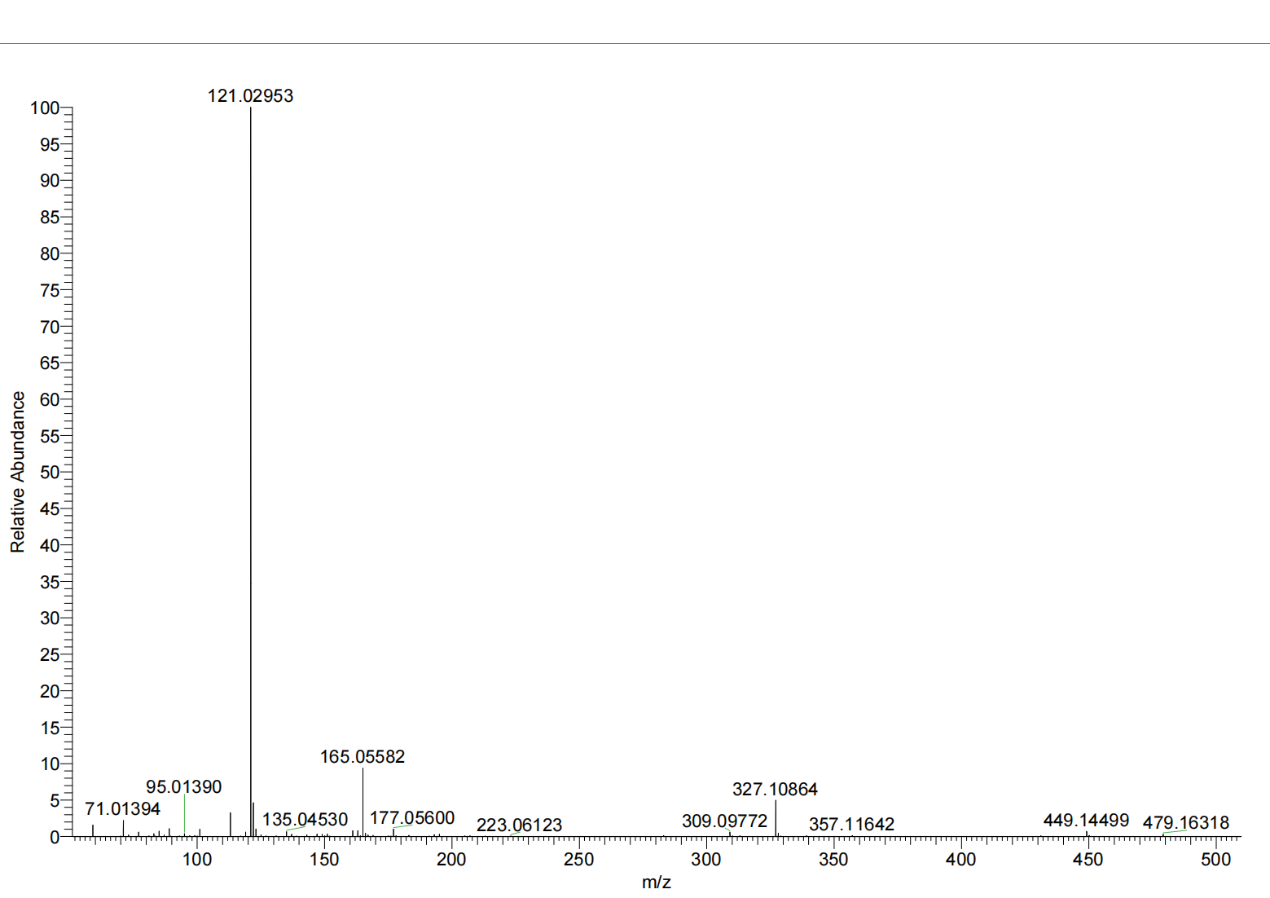


Fig. S5 MS2 Spectrum of Paeoniflorin.Compound Information: Paeoniflorin (C₂₃H₂₈O₁₁), the principal active component of Paeoniae Radix Alba in Guizhi Fuling Pills. Mass Spectrometry Parameters: Secondary mass spectrum (MS2) acquired in negative ionization mode (NEG) (consistent with Table 2 in the main text). Axis Definitions: X-axis: Mass-to-charge ratio (m/z), representing the mass of fragment ions relative to their charge. Y-axis: Relative Abundance (%), indicating the relative content of each fragment ion (normalized to the most abundant ion as 100%). Characteristic Fragment Ions: Key peaks correspond to m/z 479.1554 (molecular ion peak), 327.10864 (aglycone ion), 165.05582, 121.02953, etc.

Figure S6


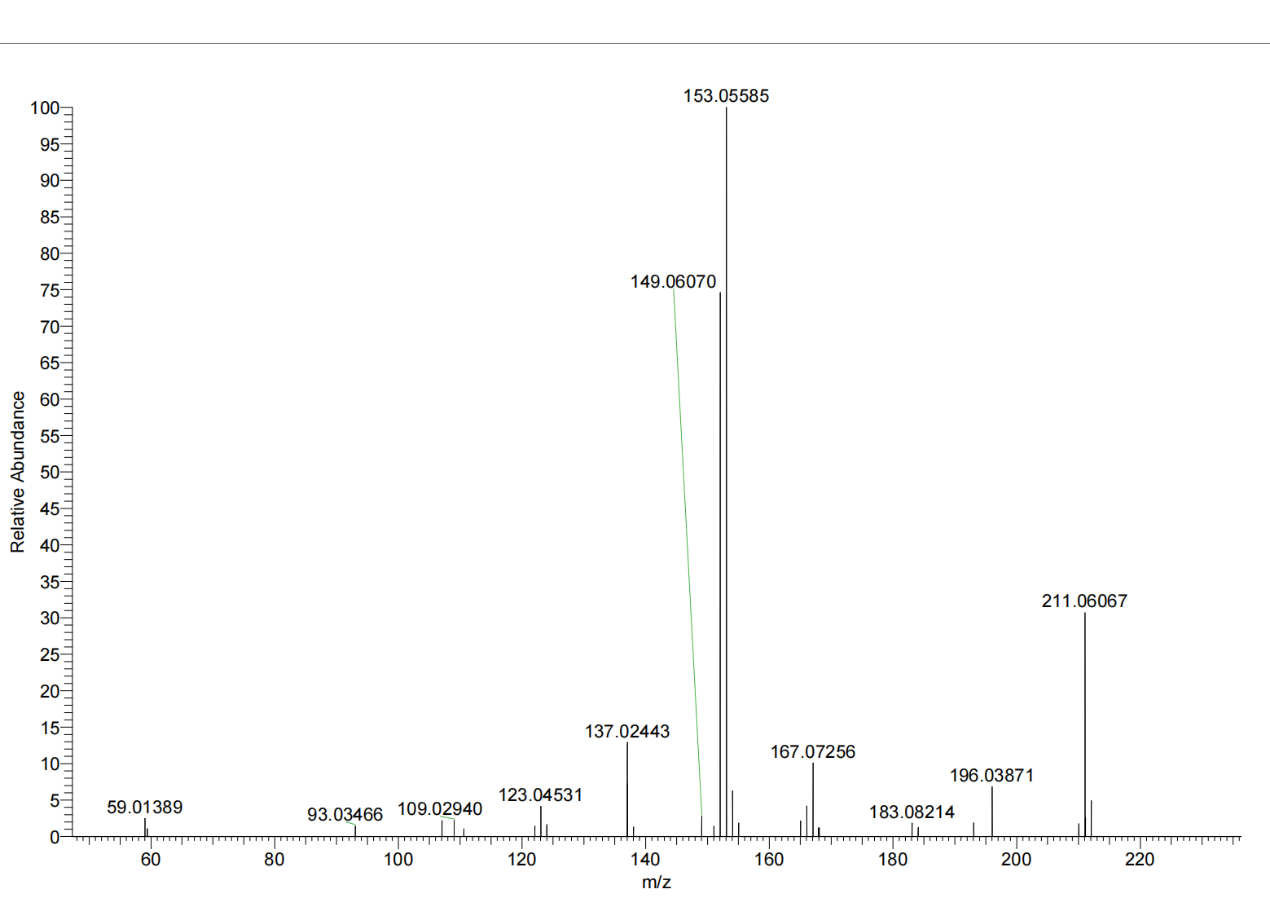


Fig. S6 MS2 Spectrum of Propylgallate. Compound Information: Propylgallate (C₁₀H₁₂O₅), a component of Guizhi Fuling Pills with antioxidant activity. Mass Spectrometry Parameters: Secondary mass spectrum (MS2) acquired in negative ionization mode (NEG) (consistent with Table 2 in the main text). Axis Definitions: X-axis: Mass-to-charge ratio (m/z), representing the mass of fragment ions relative to their charge. Y-axis: Relative Abundance (%), indicating the relative content of each fragment ion (normalized to the most abundant ion as 100%). Characteristic Fragment Ions: Key peaks correspond to m/z 211.06067 (molecular ion peak), 153.05585, 149.06070, 137.02443, 123.04531, etc., resulting from the cleavage of the propyl ester bond and the fragmentation of the gallic acid moiety.

Figure S7


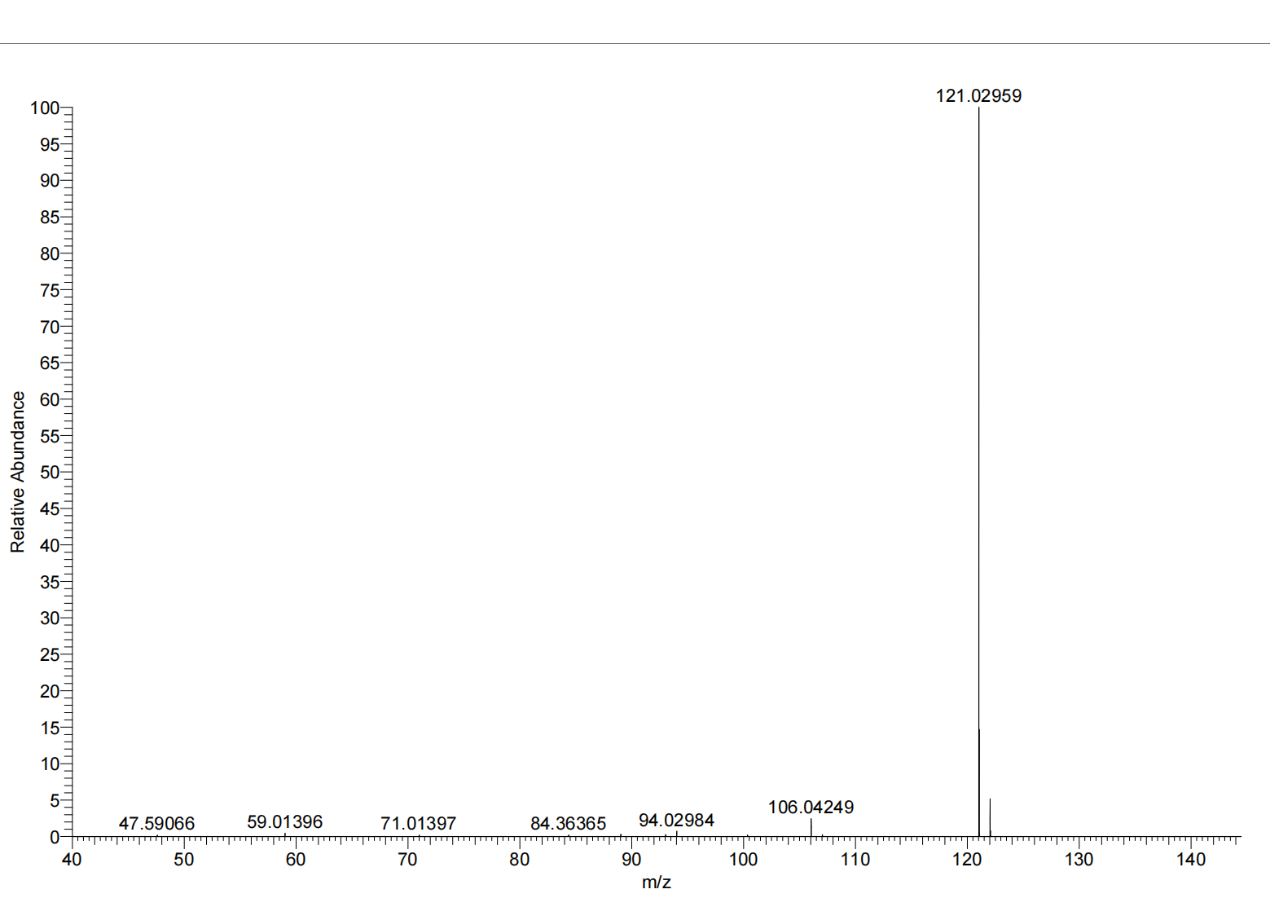


Fig. S7 MS2 Spectrum of Benzoic acid

Compound Information: Benzoic acid (C₇H₆O₂), a common component in Cinnamomi Ramulus and Poria of Guizhi Fuling Pills. Mass Spectrometry Parameters: Secondary mass spectrum (MS2) acquired in negative ionization mode (NEG) (consistent with Table 2 in the main text). Axis Definitions: X-axis: Mass-to-charge ratio (m/z), representing the mass of fragment ions relative to their charge. Y-axis: Relative Abundance (%), indicating the relative content of each fragment ion (normalized to the most abundant ion as 100%). Characteristic Fragment Ions: Key peaks correspond to m/z 121,02959 (molecular ion peak), 10604249, etc., which are the typical fragmentation features of benzoic acid (decarboxylation and benzene ring cleavage).

Figure S8


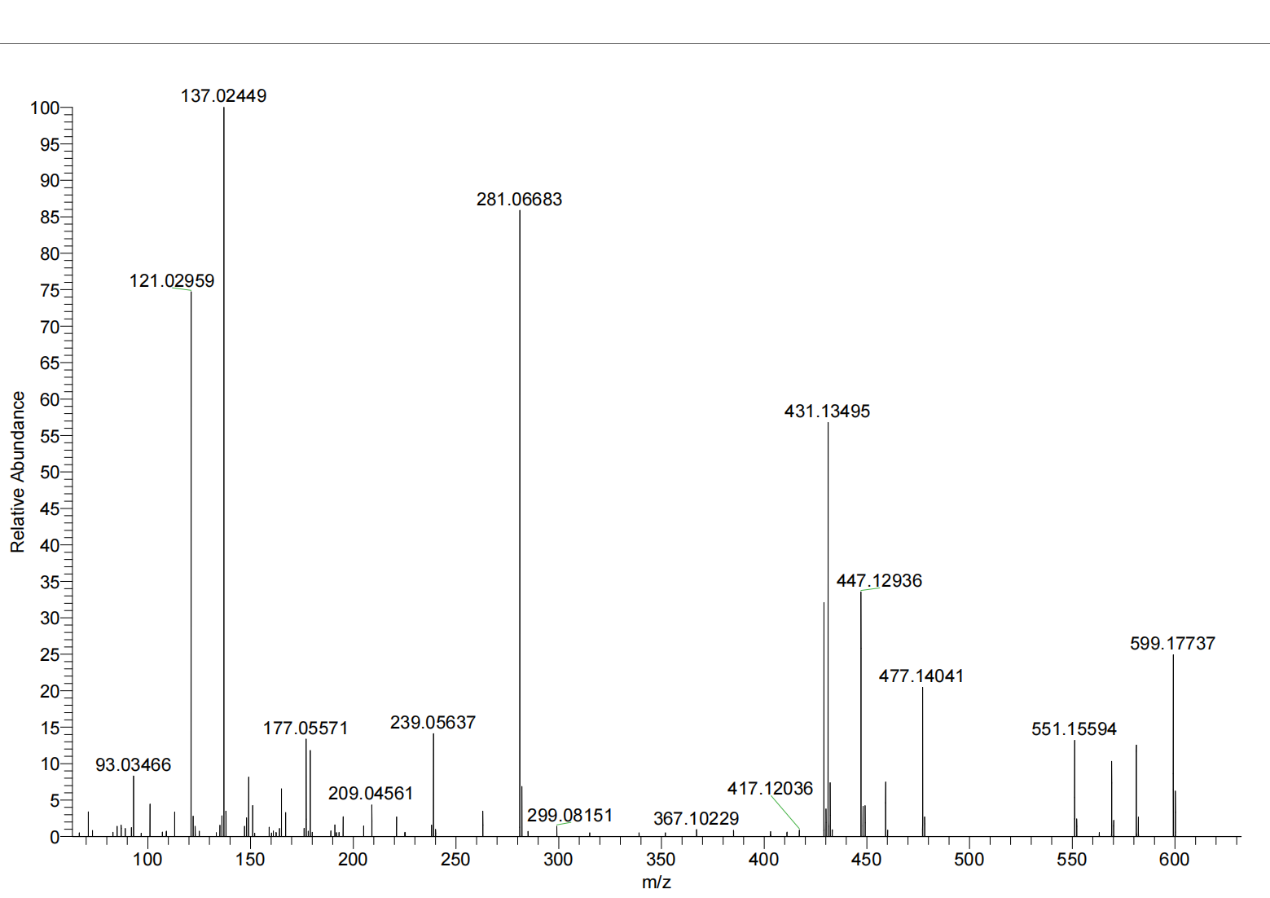


Fig. S8 MS2 Spectrum of Mudanpioside C. Compound Information: Mudanpioside C (C₃₀H₃₂O₁₃), a characteristic glycoside component of Moutan Cortex in Guizhi Fuling Pills. Mass Spectrometry Parameters: Secondary mass spectrum (MS2) acquired in negative ionization mode (NEG) (consistent with Table 2 in the main text). Axis Definitions: X-axis: Mass-to-charge ratio (m/z), representing the mass of fragment ions relative to their charge. Y-axis: Relative Abundance (%), indicating the relative content of each fragment ion (normalized to the most abundant ion as 100%). Characteristic Fragment Ions: Key peaks correspond to m/z 599.17737 (molecular ion peak), 281.06683, 137.02449, 121.02959, etc. (matching the MS2 information in Table 2 of the main text), reflecting the cleavage of sugar chains and the fragmentation of the aglycone.

Figure S9


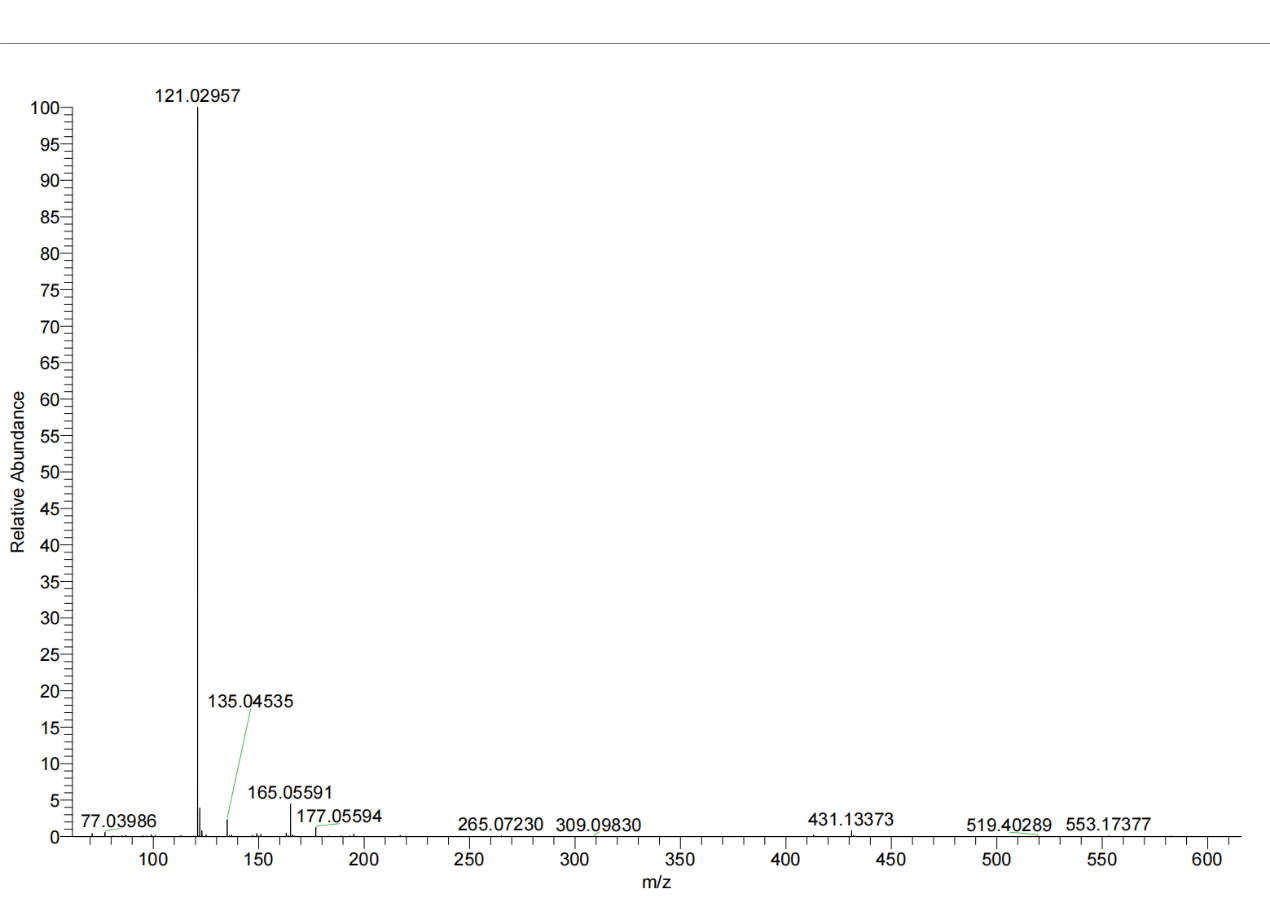


Fig. S9 MS2 Spectrum of Benzoylpaeoniflorin. Compound Information: Benzoylpaeoniflorin (C₃₀H₃₂O₁₂), a derivative of paeoniflorin in Paeoniae Radix Alba of Guizhi Fuling Pills. Mass Spectrometry Parameters: Secondary mass spectrum (MS2) acquired in negative ionization mode (NEG) (consistent with Table 2 in the main text). Axis Definitions: X-axis: Mass-to-charge ratio (m/z), representing the mass of fragment ions relative to their charge. Y-axis: Relative Abundance (%), indicating the relative content of each fragment ion (normalized to the most abundant ion as 100%). Characteristic Fragment Ions: Key peaks correspond to m/z 583.1822 (molecular ion peak), 16505591, 121.02957, etc., which are the result of benzoyl group cleavage and paeoniflorin aglycone fragmentation.

Figure S
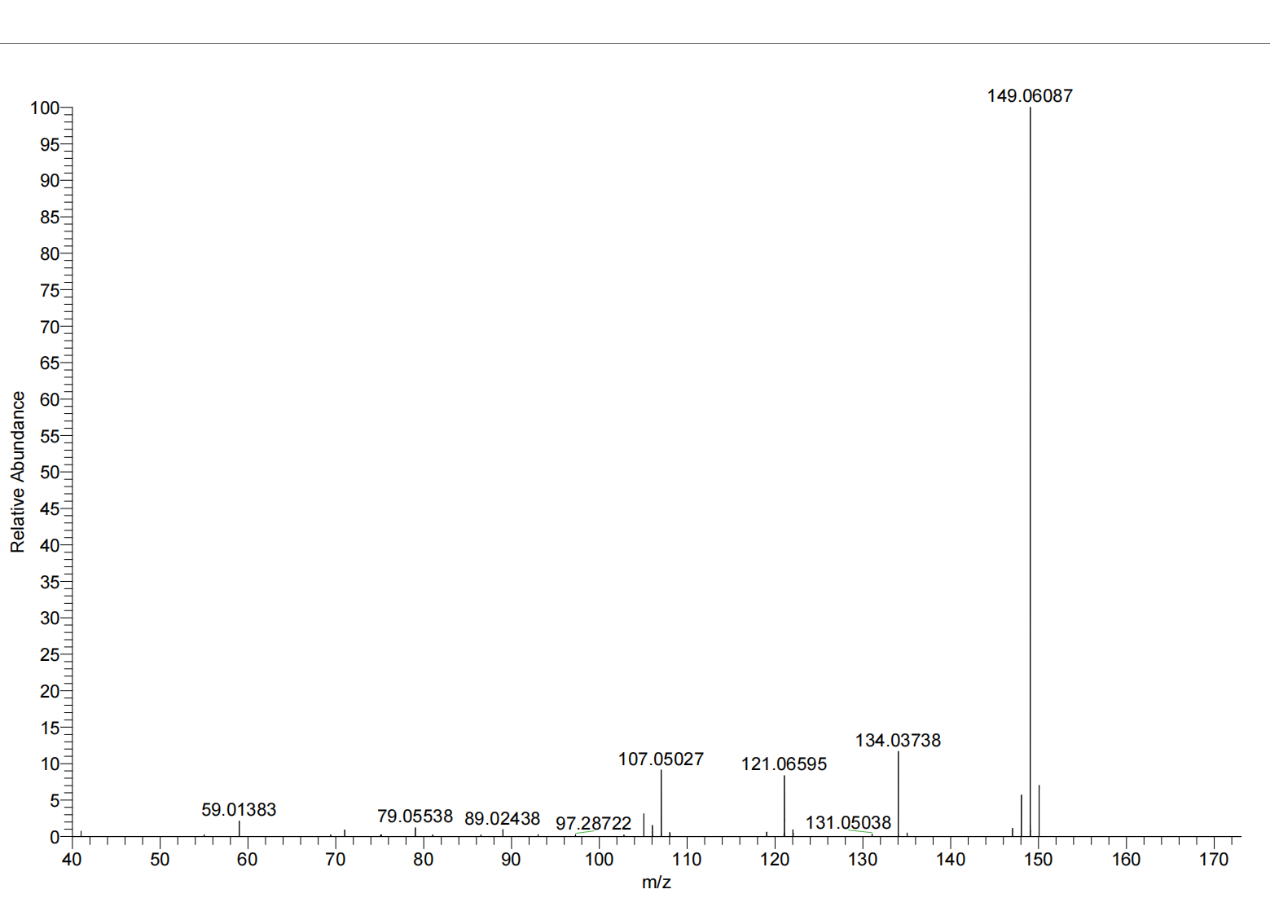
10

Fig. S10 MS2 Spectrum of 3-Phenylpropanoic acid. Compound Information: 3-Phenylpropanoic acid (C₉H₁₀O₂), an active component of Guizhi Fuling Pills with anti-inflammatory potential. Mass Spectrometry Parameters: Secondary mass spectrum (MS2) acquired in negative ionization mode (NEG) (consistent with Table 2 in the main text). Axis Definitions: X-axis: Mass-to-charge ratio (m/z), representing the mass of fragment ions relative to their charge. Y-axis: Relative Abundance (%), indicating the relative content of each fragment ion (normalized to the most abundant ion as 100%). Characteristic Fragment Ions: Key peaks correspond to m/z 149.0608 (molecular ion peak), 134.03738, 107.05027, 149.06087, etc., reflecting the cleavage of the carboxyl group and the fragmentation of the phenylpropane chain.
