# Supplementary material for: Traditional Chinese medicine prescription Guizhi Fuling Pills ameliorate cisplatin-induced renal injury via remodeling intestinal homeostasis in mice with tongue squamous cell carcinoma
Source: Front Pharmacol. 2025 Oct 2;16:1631966. doi: 10.3389/fphar.2025.1631966 (PMC12528071; doi:10.3389/fphar.2025.1631966)
Supplement: Supplementary file 2 [file Supplementaryfile2.docx]

**Table S1**

Identification of small molecular compounds in Guizhi Fuling pills

| MS2_name | Formula | mzmed | rtmed | ppm | type | MS2 |
| --- | --- | --- | --- | --- | --- | --- |
| Undecanoic acid | C11H22O2 | 185.1547 | 491.1 | 0 | NEG | 185.2;125.1;128 |
| Narirutin | C27H32O14 | 579.1719 | 291.7 | 0 | NEG | 271.1;121;151 |
| trans-11-Eicosenoic acid | C20H38O2 | 309.28 | 585.8 | 0.3 | NEG | 309.3;309.2;251.1 |
| Oleic acid | C18H34O2 | 281.2485 | 565.9 | 0.4 | NEG | 281.2 |
| trans-Vaccenic acid | C18H34O2 | 281.2485 | 565.9 | 0.4 | NEG | 281.2 |
| Continentalic acid | C20H30O2 | 301.2173 | 539.9 | 0.1 | NEG | 301.2;301;79 |
| Myristic acid | C14H28O2 | 227.2016 | 539 | 0.3 | NEG | 227.2 |
| Ursolic acid | C30H48O3 | 455.3528 | 534.1 | 0.5 | NEG | 455.4;50.6;454.3 |
| (E)-2-methyl-6-(4,4,10,13,14-pentamethyl-3,7-dioxo-2,5,6,11,12,15,16,17-octahydro-1H-cyclopenta[a]phenanthren-17-yl)hept-2-enoic acid | C30H44O4 | 467.3168 | 476.7 | 0.3 | NEG | 467.3;51.9 |
| Caprylic acid | C8H16O2 | 143.1078 | 422.2 | 0.2 | NEG | 143.1;71;85 |
| Valproic acid | C8H16O2 | 143.1078 | 422.2 | 0.2 | NEG | 143.1;71;85 |
| 1-Naphthol | C10H8O | 143.0502 | 379 | 0.1 | NEG | 143.1;71;85 |
| Royal jelly acid | C10H18O3 | 185.1183 | 344.3 | 0.2 | NEG | 185.1;125.1;139.1 |
| Tropolone | C7H6O2 | 121.0295 | 309.6 | 0.1 | NEG | 121;106 |
| Hesperidin | C28H34O15 | 609.1827 | 293.8 | 0.3 | NEG | 301.1;302.1;609.2 |
| Pyrocatechol | C6H6O2 | 109.0295 | 222.1 | 0.3 | NEG | 109;94;108 |
| Hydroquinone | C6H6O2 | 109.0295 | 222.1 | 0.3 | NEG | 109;94;108 |
| 3-methylolphenol | C7H8O2 | 123.0452 | 215.7 | 0.6 | NEG | 123;122;95 |
| (R)-5-Oxopyrrolidine-2-carboxylic acid | C5H7NO3 | 128.0353 | 61 | 0.1 | NEG | 128;85;101 |
| 1-Aminocyclopropane-1-carboxylic acid | C4H7NO2 | 100.0404 | 37.6 | 0.2 | NEG | 99;55;73 |
| 3-(2-Hydroxyphenyl)propanoic acid | C9H10O3 | 165.0557 | 310.4 | 0.1 | NEG | 121.1;165.1;147 |
| Suberic acid | C8H14O4 | 173.0819 | 292.9 | 0 | NEG | 111.1;173.1;83.1 |
| 4-Hydroxybenzaldehyde | C7H6O2 | 121.0295 | 286.4 | 0.1 | NEG | 121;77;94 |
| Vanillin | C8H8O3 | 151.04 | 273.9 | 0.1 | NEG | 136;151;137 |
| Caffeic acid | C9H8O4 | 179.035 | 262.4 | 0.5 | NEG | 135;179;136 |
| L-Arginine | C6H14N4O2 | 173.1044 | 38 | 0.1 | NEG | 131.1;173.1;102.1 |
| Pomolic acid | C30H48O4 | 471.3481 | 481.6 | 0.2 | NEG | 471.3;52.4;470.3 |
| Kaurenoic acid | C20H30O2 | 301.2173 | 539.9 | 0.1 | NEG | 301.2;301;79 |
| 1,4a,7-trimethyl-7-vinyl-3,4,6,8,8a,9,10,10a-octahydro-2H-phenanthrene-1-carboxylic acid | C20H30O2 | 301.2173 | 539.9 | 0.1 | NEG | 301.2;301;79 |
| (5R,9S)-5,9-dimethyl-14-methylene-tetracyclo[11.2.1.01,10.04,9]hexadecane-5-carboxylic acid | C20H30O2 | 301.2173 | 539.9 | 0.1 | NEG | 301.2;301;79 |
| (4aS,6aS,6bR,10S,12aR)-10-hydroxy-2,2,6a,6b,9,9,12a-heptamethyl-1,3,4,5,6,6a,7,8,8a,10,11,12,13,14b-tetradecahydropicene-4a-carboxylic acid | C30H48O3 | 455.3528 | 534.1 | 0.5 | NEG | 455.4;50.6;454.3 |
| Linoleic acid | C18H32O2 | 279.2327 | 547.4 | 0.9 | NEG | 279.2 |
| Oleamide | C18H35NO | 280.2645 | 542.6 | 0.2 | NEG | 279.2;280.2;280.3 |
| cis-9-Palmitoleic acid | C16H30O2 | 253.217 | 542.6 | 0.9 | NEG | 253.2 |
| alpha-Linolenic acid | C18H30O2 | 277.217 | 530.2 | 1.2 | NEG | 277.2;59 |
| Polyporenic acid C | C31H46O4 | 481.3323 | 488.8 | 0.1 | NEG | 481.3;53.5;421.3 |
| Capric acid | C10H20O2 | 171.1391 | 472.1 | 0.2 | NEG | 171.1 |
| Propylgallate | C10H12O5 | 211.0613 | 305 | 0.4 | NEG | 153.1;152;211.1 |
| (-)-Epicatechingallate | C22H18O10 | 441.0826 | 283.5 | 0.1 | NEG | 169;289.1;125 |
| Acetovanillone | C9H10O3 | 165.0557 | 280.5 | 0 | NEG | 150;165.1;151 |
| Homoveratric acid | C10H12O4 | 195.0662 | 275.6 | 0.2 | NEG | 136.1;195.1;123 |
| Procyanidin C1 | C45H38O18 | 865.1989 | 255.1 | 0.4 | NEG | 125;407.1;289.1 |
| Phloroglucinol | C6H6O3 | 125.0244 | 99 | 0.1 | NEG | 125;81;97 |
| Azelaic acid | C9H16O4 | 187.0975 | 325.7 | 0.3 | NEG | 125.1;187.1;97.1 |
| Succinic Acid | C4H6O4 | 117.0193 | 76.9 | 0.1 | NEG | 73;117;116.9 |
| (-)-Quinicacid | C7H12O6 | 191.056 | 43.1 | 0.3 | NEG | 191.1;85;111 |
| Benzoylpaeoniflorin | C30H32O12 | 583.1822 | 348.3 | 0.2 | NEG | 121;165.1;122 |
| (4aS,6aS,6bR,9R,10S,12aR,14bS)-10-hydroxy-9-(hydroxymethyl)-2,2,6a,6b,9,12a-hexamethyl-1,3,4,5,6,6a,7,8,8a,10,11,12,13,14b-tetradecahydropicene-4a-carboxylic acid | C30H48O4 | 471.3481 | 481.6 | 0.2 | NEG | 471.3;52.4;470.3 |
| (1S,4aR,10aR)-1,4a,7-trimethyl-7-vinyl-3,4,4b,5,6,9,10,10a-octahydro-2H-phenanthrene-1-carboxylic acid | C20H30O2 | 301.2173 | 539.9 | 0.1 | NEG | 301.2;301;79 |
| gamma-Linolenic acid | C18H30O2 | 277.217 | 530.2 | 1.2 | NEG | 277.2;59 |
| (2R)-2-[(3S,5R,10S,13R,14R,17R)-3-hydroxy-4,4,10,13,14-pentamethyl-2,3,5,6,7,11,12,15,16,17-decahydro-1H-cyclopenta[a]phenanthren-17-yl]-6-methyl-5-methylene-heptanoic acid | C31H50O3 | 469.3688 | 562.7 | 0.2 | NEG | 469.4;52.2;169 |
| Pentadecanoic acid | C15H30O2 | 241.2172 | 552.3 | 0.2 | NEG | 241.2;225.1;181.2 |
| (E)-5-(1,2,4a,5-tetramethyl-2,3,4,7,8,8a-hexahydronaphthalen-1-yl)-3-methyl-pent-2-enoic acid | C20H32O2 | 303.2328 | 541.7 | 0.5 | NEG | 303.2;302.2;59 |
| 12-Hydroxystearic acid | C18H36O3 | 299.2591 | 513.7 | 0 | NEG | 299.3;298.2;237.2 |
| 10-Undecenoic acid | C11H20O2 | 183.139 | 468.9 | 0 | NEG | 183.1;101;146.5 |
| Pelargonic acid | C9H18O2 | 157.1233 | 450.8 | 0.4 | NEG | 157.1;100.9 |
| Kaempferol | C15H10O6 | 285.0406 | 368.8 | 0.6 | NEG | 285;121;284.3 |
| 2-Hydroxyhexanoic acid | C6H12O3 | 131.0713 | 257.5 | 0 | NEG | 131.1;85.1;86.1 |
| Apiopaeonoside | C20H28O12 | 459.1509 | 257.2 | 0.2 | NEG | 165.1;459.2;121.1 |
| (2Z,4E)-5-[(1R,3R,5S,8S)-3,8-dihydroxy-1,5-dimethyl-6-oxabicyclo[3.2.1]octan-8-yl]-3-methyl-penta-2,4-dienoic acid | C15H22O5 | 281.1395 | 252.7 | 0.4 | NEG | 281.1;237.1;171.1 |
| Catechin | C15H14O6 | 289.0717 | 241.2 | 0.2 | NEG | 289.1;245.1;125 |
| DL-Tryptophan | C11H12N2O2 | 203.0827 | 205.5 | 0.4 | NEG | 116.1;203.1;74 |
| (2R)-2-phenyl-2-[(2S,3R,4S,5S,6R)-3,4,5-trihydroxy-6-(hydroxymethyl)tetrahydropyran-2-yl]oxy-acetic acid | C14H18O8 | 313.0929 | 156.3 | 0.2 | NEG | 101;71;59 |
| Malic acid | C4H6O5 | 133.0141 | 59.9 | 0.9 | NEG | 115;133;71 |
| Chiro-Inositol | C6H12O6 | 179.056 | 40.7 | 0.7 | NEG | 179.1;87;161 |
| Myo-Inositol | C6H12O6 | 179.056 | 40.7 | 0.7 | NEG | 179.1;87;161 |
| Gentisic acid | C7H6O4 | 153.0194 | 218.7 | 0.5 | NEG | 109;153;108 |
| Anemosapogenin | C30H48O4 | 471.3481 | 481.6 | 0.2 | NEG | 471.3;52.4;470.3 |
| Benzoic acid | C7H6O2 | 121.0295 | 309.6 | 0.2 | NEG | 121;106 |
| ethyl octanoate | C10H20O2 | 171.1391 | 472.1 | 0.2 | NEG | 171.1 |
| Quinic acid | C7H12O6 | 191.056 | 43.1 | 0.3 | NEG | 191.1;85;111 |
| Arachidonic acid (AA) | C20H32O2 | 303.2328 | 541.7 | 0.5 | NEG | 303.2;302.2;59 |
| Hydroxyisocaproic acid | C6H12O3 | 131.0713 | 257.5 | 0 | NEG | 131.1;85.1;86.1 |
| H-D-Trp-OH | C11H12N2O2 | 203.0827 | 205.5 | 0.4 | NEG | 116.1;203.1;74 |
| Palmitic acid | C16H32O2 | 255.2329 | 563.6 | 0.2 | NEG | 255.2 |
| Methyl pentadecanoate | C16H32O2 | 255.2329 | 563.6 | 0.2 | NEG | 255.2 |
| Dodecanoic acid | C12H24O2 | 199.1703 | 509 | 0.3 | NEG | 199.2 |
| (E)-5-(2,3-dimethyl-3-tricyclo[2.2.1.02,6]heptanyl)-2-methyl-pent-2-enoic acid | C15H22O2 | 233.1547 | 488.4 | 0.1 | NEG | 233.2;97 |
| 16-Hydroxypalmitic acid | C16H32O3 | 271.2278 | 478.1 | 0.1 | NEG | 271.2;94.9;92.9 |
| (4aR,5R,6aR,6aS,6bR,8aR,9R,10S,12aR,14bS)-5,10-dihydroxy-9-(hydroxymethyl)-2,2,6a,6b,9,12a-hexamethyl-1,3,4,5,6,6a,7,8,8a,10,11,12,13,14b-tetradecahydropicene-4a-carboxylic acid | C30H48O5 | 487.3434 | 445.9 | 1.1 | NEG | 487.3;54.1;94.9 |
| trans-2-Octenoic acid | C8H14O2 | 141.0921 | 415 | 0.2 | NEG | 59;141.1;141 |
| Paeoniflorin | C23H28O11 | 479.1554 | 268.6 | 0.9 | NEG | 121;165.1;327.1 |
| 2-methylcitrate | C7H10O7 | 205.0352 | 44.4 | 0.6 | NEG | 125;205;81 |
| Glyceraldehyde | C3H6O3 | 89.0244 | 40.3 | 0.5 | NEG | 89;59;43 |
| Sebacic acid | C10H18O4 | 201.1132 | 353.2 | 0.2 | NEG | 139.1;201.1;183.1 |
| Stearic acid | C18H36O2 | 283.2642 | 586.3 | 0 | NEG | 283.3 |
| Procyanidin B2 | C30H26O12 | 579.1482 | 243.8 | 2.7 | POS | 127;139;287.1 |
| Albiflorin | C23H28O11 | 479.1557 | 253.5 | 0.3 | NEG | 121;122 |
| [(1S,2S,3R,5R,6S,8S)-6-hydroxy-8-methyl-3-[(2S,3R,4S,5S,6R)-3,4,5-trihydroxy-6-(hydroxymethyl)tetrahydropyran-2-yl]oxy-9,10-dioxatetracyclo[4.3.1.02,5.03,8]decan-2-yl]methyl benzoate | C23H28O11 | 479.1557 | 253.5 | 0.3 | NEG | 121;122 |
| 12-Methyltridecanoic acid | C14H28O2 | 227.2016 | 539 | 0.3 | NEG | 227.2 |
| Pyroglutamic acid | C5H7NO3 | 128.0353 | 61 | 0.1 | NEG | 128;85;101 |
| 3-Hydroxybenzaldehyde | C7H6O2 | 121.0295 | 286.4 | 0.1 | NEG | 121;77;94 |
| Pyrogallol | C6H6O3 | 125.0244 | 99 | 0.1 | NEG | 125;81;97 |
| hexyl hexanoate | C12H24O2 | 199.1703 | 509 | 0.3 | NEG | 199.2 |
| Euscaphic acid | C30H48O5 | 487.3434 | 445.9 | 1.1 | NEG | 487.3;54.1;94.9 |
| (1S,2R,4aS,6aS,6bR,9R,10R,11R,12aR)-10,11-dihydroxy-9-(hydroxymethyl)-1,2,6a,6b,9,12a-hexamethyl-2,3,4,5,6,6a,7,8,8a,10,11,12,13,14b-tetradecahydro-1H-picene-4a-carboxylic acid | C30H48O5 | 487.3434 | 445.9 | 1.1 | NEG | 487.3;54.1;94.9 |
| Heptadecanoic acid | C17H34O2 | 269.2486 | 575.8 | 0.1 | NEG | 269.2;123.9;64.4 |
| Poricoic acid A | C31H46O5 | 497.3273 | 492 | 0.1 | NEG | 497.3;423.3;424.3 |
| 13(S)-HODE | C18H32O3 | 295.2277 | 482 | 0.4 | NEG | 295.2;277.2;294.2 |
| 14,16-dihydroxy-4-methyl-3-oxabicyclo[10.4.0]hexadeca-1(12),13,15-trien-2-one | C16H22O4 | 277.1445 | 477.1 | 0 | NEG | 277.1;233.2;259.1 |
| 14-hydroxy-16-methoxy-4-methyl-3-oxabicyclo[10.4.0]hexadeca-1(12),13,15-trien-2-one | C17H24O4 | 291.1603 | 444.9 | 0.6 | NEG | 291.2;247.2;247 |
| 2-[(2S,4aR,8aS)-2-hydroxy-4a-methyl-8-methylene-decalin-2-yl]prop-2-enoic acid | C15H22O3 | 249.1497 | 441.1 | 0.3 | NEG | 249.1;231.1;185.1 |
| 7-(1-hydroxy-1-methyl-ethyl)-1,4a-dimethyl-2,3,4,9,10,10a-hexahydrophenanthrene-1-carboxylic acid | C20H28O3 | 315.1967 | 431.4 | 0.5 | NEG | 315.2;300;314.2 |
| 4-(4-methoxyphenyl)butan-2-one | C11H14O2 | 177.0921 | 427.4 | 0.4 | NEG | 177.1;149.1;59 |
| ethyl 2,2-dimethyl-3-(2-methylprop-1-enyl)cyclopropanecarboxylate | C12H20O2 | 195.1391 | 423.3 | 0.3 | NEG | 195.1;167.1;87 |
| 3-phenyl-1-(2,4,6-trihydroxyphenyl)prop-2-en-1-one | C15H12O4 | 255.066 | 401.9 | 1.1 | NEG | 255.1;213.1;151 |
| Caproic acid | C6H12O2 | 115.0765 | 363.1 | 0.4 | NEG | 115.1;71;99.9 |
| 4-Allylcatechol | C9H10O2 | 149.0608 | 352.8 | 0.1 | NEG | 149.1;134;107.1 |
| 3-Hydroxyoctanoic acid | C8H16O3 | 159.1027 | 351.6 | 0.1 | NEG | 59;100.9;159.1 |
| Salicylic acid | C7H6O3 | 137.0244 | 300.4 | 0.1 | NEG | 93;137;94 |
| Neoamygdalin | C20H27NO11 | 456.1508 | 239.9 | 0.7 | NEG | 89;59;101 |
| Phenylalanine | C9H11NO2 | 164.0717 | 97.3 | 0.4 | NEG | 147;164.1;72 |
| Hydroxyacetone | C3H6O2 | 73.0295 | 63.8 | 0.1 | NEG | 73;45;72 |
| 3,4-Dihydroxybenzoic acid | C7H6O4 | 153.0193 | 205.7 | 0.2 | NEG | 109;153;108 |
| D-Pantothenic Acid | C9H17NO5 | 218.1034 | 180.1 | 0.2 | NEG | 88;146.1;218.1 |
| DEHP | C24H38O4 | 391.2832 | 564.5 | 2.8 | POS | 149;71.1;57.1 |
| Epicatechin | C15H14O6 | 291.0855 | 256.4 | 2.9 | POS | 139;123;165.1 |
| Guanosine | C10H13N5O5 | 284.0982 | 94.1 | 2.6 | POS | 152.1;266.1;70.1 |
| Adenine | C5H5N5 | 136.0613 | 68.4 | 3.3 | POS | 136.1;81.1;89.1 |
| Leucine | C6H13NO2 | 132.1014 | 62.3 | 3.6 | POS | 86.1;132.1;87.1 |
| Allose | C6H12O6 | 203.052 | 39.9 | 3.5 | POS | 203.1;202.2;60 |
| Echinocystic acid | C30H48O4 | 471.3481 | 481.6 | 0.2 | NEG | 471.3;52.4;470.3 |
| (1R,2R,4aS,6aS,6bR,10S,12aR)-1,10-dihydroxy-1,2,6a,6b,9,9,12a-heptamethyl-2,3,4,5,6,6a,7,8,8a,10,11,12,13,14b-tetradecahydropicene-4a-carboxylic acid | C30H48O4 | 471.3481 | 481.6 | 0.2 | NEG | 471.3;52.4;470.3 |
| Paullinic acid | C20H38O2 | 309.28 | 585.8 | 0.3 | NEG | 309.3;309.2;251.1 |
| Isopimaric acid | C20H30O2 | 301.2173 | 539.9 | 0.1 | NEG | 301.2;301;79 |
| Hesperetin 7-neohesperidoside | C28H34O15 | 609.1827 | 293.8 | 0.3 | NEG | 301.1;302.1;609.2 |
| Fructose | C6H12O6 | 179.056 | 40.7 | 0.7 | NEG | 179.1;87;161 |
| 2,3-Dihydroxybenzoic acid | C7H6O4 | 153.0194 | 218.7 | 0.5 | NEG | 109;153;108 |
| (4aS,6aS,6bR,9R,10R,11R,12aR)-10,11-dihydroxy-9-(hydroxymethyl)-2,2,6a,6b,9,12a-hexamethyl-1,3,4,5,6,6a,7,8,8a,10,11,12,13,14b-tetradecahydropicene-4a-carboxylic acid | C30H48O5 | 487.3434 | 445.9 | 1.1 | NEG | 487.3;54.1;94.9 |
| (1R,2R,4aS,6aS,6bR,10S,11R,12aR)-1,10,11-trihydroxy-1,2,6a,6b,9,9,12a-heptamethyl-2,3,4,5,6,6a,7,8,8a,10,11,12,13,14b-tetradecahydropicene-4a-carboxylic acid | C30H48O5 | 487.3434 | 445.9 | 1.1 | NEG | 487.3;54.1;94.9 |
| 3-Phenylpropanoic acid | C9H10O2 | 149.0608 | 352.8 | 0.1 | NEG | 149.1;134;107.1 |
| Amygdalin | C20H27NO11 | 456.1508 | 239.9 | 0.7 | NEG | 89;59;101 |
| 9-Oxo-10(E),12(E)-octadecadienoic acid | C18H30O3 | 293.2121 | 479.8 | 0.4 | NEG | 293.2;185.1;125.1 |
| Dehydrotumulosic acid | C31H48O4 | 483.3474 | 479.8 | 1.1 | NEG | 483.3;53.7;409.3 |
| Emodin | C15H10O5 | 269.0456 | 452.6 | 0.4 | NEG | 269;225.1;92.9 |
| (Z)-9,10,11-trihydroxyoctadec-12-enoic acid | C18H34O5 | 329.2335 | 405 | 0.7 | NEG | 329.2;199.1;211.1 |
| 2',6'-Dihydroxyacetophenone | C8H8O3 | 151.0401 | 313.3 | 0.3 | NEG | 151;109;135 |
| 4'-Hydroxy-3'-methylacetophenone | C9H10O2 | 149.0608 | 307.1 | 0.1 | NEG | 149.1;131.1;43 |
| Baimaside | C27H30O17 | 625.1419 | 275.5 | 1.5 | NEG | 300;625.1;301 |
| Methylgallate | C8H8O5 | 183.0299 | 243.7 | 0.1 | NEG | 183;124;168 |
| Succinyladenosine | C14H17N5O8 | 382.1006 | 210.9 | 0.5 | NEG | 134;206.1;382.1 |
| Galactaric acid | C6H10O8 | 209.0302 | 60.6 | 0.2 | NEG | 85;209;191 |
| Fructose 6-phosphate | C6H13O9P | 259.0222 | 57.6 | 0.8 | NEG | 97;79;259 |
| 3-Hydroxypropionic acid (beta-lactic acid) | C3H6O3 | 89.0244 | 50.4 | 0.4 | NEG | 89;59;43 |
| Glyceric acid | C3H6O4 | 105.0192 | 43.4 | 0.7 | NEG | 105;75;59 |
| Galactose | C6H12O6 | 203.052 | 39.9 | 3.5 | POS | 203.1;202.2;60 |
| Asperphenamate | C32H30N2O4 | 507.2267 | 442.3 | 2.3 | POS | 238.1;105;224.1 |
| (±)-Naringenin | C15H12O5 | 273.0748 | 355.9 | 3.4 | POS | 273.1;153;147 |
| 3-Hydroxy-4-methoxyacetophenone | C9H10O3 | 167.0697 | 247.8 | 3.4 | POS | 167.1;43;149.1 |
| Phe-Leu | C15H22N2O3 | 279.1695 | 246.9 | 3.1 | POS | 120.1;279.1;86.1 |
| 6-(hydroxymethyl)pyridin-3-ol | C6H7NO2 | 126.0545 | 58.4 | 3.6 | POS | 108;109;126.1 |
| D(+)-Pipecolinic acid | C6H11NO2 | 130.0858 | 44.5 | 3.6 | POS | 84.1;130.1;70.1 |
| Pipecolic acid | C6H11NO2 | 130.0858 | 44.5 | 3.6 | POS | 84.1;130.1;70.1 |
| Hygric acid | C6H11NO2 | 130.0858 | 44.5 | 3.6 | POS | 84.1;130.1;70.1 |
| 5-Hydroxymethylfurfural | C6H6O3 | 127.0386 | 93.3 | 3.4 | POS | 127;109;69 |
| Adenosine | C10H13N5O4 | 268.1035 | 202.9 | 2.1 | POS | 136.1;268.1;267.1 |
| 7-[4,5-dihydroxy-6-(hydroxymethyl)-3-[(2S,3R,4R,5R,6S)-3,4,5-trihydroxy-6-methyl-tetrahydropyran-2-yl]oxy-tetrahydropyran-2-yl]oxy-5-hydroxy-2-(4-hydroxyphenyl)chroman-4-one | C27H32O14 | 579.1719 | 291.7 | 0 | NEG | 271.1;121;151 |
| Naringin | C27H32O14 | 579.1719 | 291.7 | 0 | NEG | 271.1;121;151 |
| [(2R,3S,4S,5R,6S)-6-[[(1S,3R,5R,6S,8S)-2-(benzoyloxymethyl)-6-hydroxy-8-methyl-9,10-dioxatetracyclo[4.3.1.02,5.03,8]decan-3-yl]oxy]-3,4,5-trihydroxy-tetrahydropyran-2-yl]methyl benzoate | C30H32O12 | 583.1822 | 348.3 | 0.2 | NEG | 121;165.1;122 |
| (E)-8-hydroxy-2,6-dimethyl-oct-2-enoic acid | C10H18O3 | 185.1183 | 344.3 | 0.2 | NEG | 185.1;125.1;139.1 |
| Hesperetin 7-O-neohesperidoside | C28H34O15 | 609.1827 | 293.8 | 0.3 | NEG | 301.1;302.1;609.2 |
| 5,7-dihydroxy-2-phenyl-chroman-4-one | C15H12O4 | 255.066 | 401.9 | 1.1 | NEG | 255.1;213.1;151 |
| Myristoleic acid | C14H26O2 | 225.186 | 517.8 | 0 | NEG | 225.2;127.5;51.8 |
| 9-Oxooctadecanoic acid | C18H34O3 | 297.2435 | 516 | 0.1 | NEG | 297.2;279.2;171.1 |
| Octadecanedioic acid | C18H34O4 | 313.2384 | 500.6 | 0.1 | NEG | 313.2;251.2;295.2 |
| Dodecanedioic acid | C12H22O4 | 211.134 | 392.9 | 0.5 | NEG | 211.1;183.1;167.1 |
| (E,9S,12S,13S)-9,12,13-trihydroxyoctadec-10-enoic acid | C18H34O5 | 329.2333 | 391.6 | 0.1 | NEG | 329.2;211.1;229.1 |
| 4-hydroxy-3-(3-methylbut-2-enyl)benzoic acid | C12H14O3 | 205.0871 | 387.2 | 0.3 | NEG | 161.1;205.1;106 |
| Pimelic acid | C7H12O4 | 159.0663 | 248.2 | 0.1 | NEG | 97.1;159.1;115.1 |
| D-(-)-Mandelic acid | C8H8O3 | 151.0401 | 195 | 0.1 | NEG | 107.1;151;89 |
| DL-Mandelic acid | C8H8O3 | 151.0401 | 195 | 0.1 | NEG | 107.1;151;89 |
| 2-hydroxy-5-[(2S,3R,4S,5S,6R)-3,4,5-trihydroxy-6-(hydroxymethyl)tetrahydropyran-2-yl]oxy-benzoic acid | C13H16O9 | 315.0722 | 113.3 | 0.2 | NEG | 315.1;152;108 |
| Sucrose | C12H22O11 | 341.1085 | 48.2 | 1.2 | NEG | 89;59;71 |
| Sorbose | C6H12O6 | 203.052 | 39.9 | 3.5 | POS | 203.1;202.2;60 |
| Xanyhyletin | C14H12O3 | 229.0851 | 401.3 | 3.6 | POS | 229.1;155.1;101 |
| Prostaglandin E1 (PGE1) | C20H34O5 | 377.2287 | 395.9 | 3.2 | POS | 377.2;332.2;359.2 |
| Hinokitiol | C10H12O2 | 165.0904 | 331.9 | 3.7 | POS | 165.1;150.1;137.1 |
| Tryptophan | C11H12N2O2 | 205.0966 | 200.8 | 2.7 | POS | 188.1;146.1;144.1 |
| Isonicotinic acid | C6H5NO2 | 124.0389 | 49 | 3.7 | POS | 124;123;119 |
| Paeonolide | C20H28O12 | 459.1509 | 257.2 | 0.2 | NEG | 165.1;459.2;121.1 |
| (1S,4aR,6aS,6bR,10R,11R,12aR,14bS)-1,10,11-trihydroxy-2,2,6a,6b,9,9,12a-heptamethyl-1,3,4,5,6,6a,7,8,8a,10,11,12,13,14b-tetradecahydropicene-4a-carboxylic acid | C30H48O5 | 487.3434 | 445.9 | 1.1 | NEG | 487.3;54.1;94.9 |
| 4′-Hydroxy-2′-methylacetophenone | C9H10O2 | 149.0608 | 307.1 | 0.1 | NEG | 149.1;131.1;43 |
| Glucose 6-phosphate | C6H13O9P | 259.0222 | 57.6 | 0.8 | NEG | 97;79;259 |
| Lactate | C3H6O3 | 89.0244 | 50.4 | 0.4 | NEG | 89;59;43 |
| Trehalose | C12H22O11 | 341.1085 | 48.2 | 1.2 | NEG | 89;59;71 |
| (2S,4aS,6aS,6bR,10S,12aS,14bS)-10-hydroxy-2,4a,6a,6b,9,9,12a-heptamethyl-13-oxo-3,4,5,6,6a,7,8,8a,10,11,12,14b-dodecahydro-1H-picene-2-carboxylic acid | C30H46O4 | 469.3323 | 480.7 | 0 | NEG | 469.3;439.3;367.3 |
| Hexadecanedioic acid | C16H30O4 | 285.2072 | 470.7 | 0.3 | NEG | 285.2;267.2;223.2 |
| (S)-Abscisic acid | C15H20O4 | 263.1289 | 332.9 | 0.3 | NEG | 153.1;219.1;204.1 |
| 2-phenyl-2-[3,4,5-trihydroxy-6-(hydroxymethyl)tetrahydropyran-2-yl]oxy-acetonitrile | C14H17NO6 | 294.0983 | 258.4 | 0.1 | NEG | 71;161;101 |
| Prunasin | C14H17NO6 | 294.0983 | 258.4 | 0.1 | NEG | 71;161;101 |
| 3-Hydroxybenzoic acid | C7H6O3 | 137.0244 | 248.8 | 0.1 | NEG | 93;137;94 |
| (2R,3R,4S,5S,6R)-2-[[(2R,3S,4S,5R,6R)-6-benzyloxy-3,4,5-trihydroxy-tetrahydropyran-2-yl]methoxy]-6-(hydroxymethyl)tetrahydropyran-3,4,5-triol | C19H28O11 | 431.1559 | 232.8 | 0.1 | NEG | 101;71;59 |
| 3,4-Dihydroxyphenylacetic acid | C8H8O4 | 149.0245 | 225.1 | 0.8 | NEG | 148.1;149;104.1 |
| Threonic acid | C4H8O5 | 135.0298 | 42.6 | 0.8 | NEG | 75;135;59 |
| Arachidic acid | C20H40O2 | 311.2955 | 607.3 | 0.1 | NEG | 311.2;311.3;183 |
| Norleucine | C6H13NO2 | 132.1014 | 62.3 | 3.6 | POS | 86.1;132.1;87.1 |
| 2-Hydroxy-4-methylbenzaldehyde | C8H8O2 | 137.0591 | 351 | 4.3 | POS | 137.1;43;81.1 |
| naphthalene-1,3-diol | C10H8O2 | 161.0591 | 334.4 | 3.9 | POS | 161.1;105.1;133.1 |
| 2',4',6'-Trihydroxyacetophenone | C8H8O4 | 151.0384 | 316.4 | 3.4 | POS | 151;95.1;123.1 |
| (-)-Catechin | C15H14O6 | 291.0853 | 240.4 | 3.4 | POS | 139;123;165.1 |
| Nicotinamide | C6H6N2O | 123.0549 | 88.5 | 3.1 | POS | 123.1;105.1;80 |
| Nicotinate | C6H5NO2 | 124.0389 | 61.5 | 3.7 | POS | 124;97;119 |
| 3-Epiursolic Acid | C30H48O3 | 455.3528 | 534.1 | 0.5 | NEG | 455.4;50.6;454.3 |
| 3-Phenyllactic acid | C9H10O3 | 165.0557 | 280.5 | 0 | NEG | 150;165.1;151 |
| D-Mannoheptulose | C7H14O7 | 191.056 | 43.1 | 0.1 | NEG | 191.1;85;111 |
| (1S,2R,4aS,6aS,6bR,9R,10S,11R,12aR,14bS)-10,11-dihydroxy-9-(hydroxymethyl)-1,2,6a,6b,9,12a-hexamethyl-2,3,4,5,6,6a,7,8,8a,10,11,12,13,14b-tetradecahydro-1H-picene-4a-carboxylic acid | C30H48O5 | 487.3434 | 445.9 | 1.1 | NEG | 487.3;54.1;94.9 |
| Dihydroxyacetone | C3H6O3 | 89.0244 | 50.4 | 0.4 | NEG | 89;59;43 |
| 4-Hydroxybenzoic acid | C7H6O3 | 137.0244 | 248.8 | 0.1 | NEG | 93;137;94 |
| 2-Hydroxydecanoic-acid | C10H20O3 | 187.134 | 437.9 | 0.3 | NEG | 141.1;187.1;125.1 |
| (2R,3R,4S,5S,6R)-2-[(2E)-3,7-dimethylocta-2,6-dienoxy]-6-[[(2S,3R,4S,5S)-3,4,5-trihydroxytetrahydropyran-2-yl]oxymethyl]tetrahydropyran-3,4,5-triol | C21H36O10 | 447.2236 | 349.1 | 0.1 | NEG | 447.2;315.2;101 |
| Quercetin | C15H10O7 | 301.0355 | 344.5 | 0.7 | NEG | 301;151;301 |
| (2R,3R,4S,5S,6R)-2-benzyloxy-6-(hydroxymethyl)tetrahydropyran-3,4,5-triol | C13H18O6 | 269.1032 | 246.7 | 0.5 | NEG | 101;71;59 |
| Terephthalic-Acid | C8H6O4 | 165.0194 | 246.3 | 0.3 | NEG | 121;93;165 |
| (4R,4aR,6R,7R,7aS)-4,7-dimethyl-6-[(2R,3R,4S,5S,6R)-3,4,5-trihydroxy-6-(hydroxymethyl)tetrahydropyran-2-yl]oxy-4,4a,5,6,7,7a-hexahydro-3H-cyclopenta[c]pyran-1-one | C16H26O8 | 345.1554 | 234.6 | 0.1 | NEG | 59;89;101 |
| [(1R,21S,23R)-6,7,8,11,12,13,22,23-octahydroxy-3,16-dioxo-2,17,20-trioxatetracyclo[17.3.1.04,9.010,15]tricosa-4,6,8,10,12,14-hexaen-21-yl] 3,4,5-trihydroxybenzoate | C27H22O18 | 633.074 | 232.8 | 1.1 | NEG | 301;633.1;275 |
| Orotic acid | C5H4N2O4 | 155.0098 | 63 | 0.2 | NEG | 111;111;83 |
| Pyruvate | C3H4O3 | 87.0087 | 61.8 | 0.3 | NEG | 87;41;59 |
| N-Acetylphenylalanine | C11H13NO3 | 206.0823 | 280.1 | 0.1 | NEG | 164.1;58;91.1 |
| Isoleucine | C6H13NO2 | 132.1014 | 62.3 | 3.6 | POS | 86.1;132.1;87.1 |
| 8,8-dimethylpyrano[2,3-f]chromen-2-one | C14H12O3 | 229.0851 | 401.3 | 3.6 | POS | 229.1;155.1;101 |
| 4-[5-(4-hydroxy-3-methoxy-phenyl)-3,4-dimethyl-tetrahydrofuran-2-yl]-2-methoxy-phenol | C20H24O5 | 345.1686 | 383.8 | 3.2 | POS | 165.1;137.1;221.1 |
| 4-Methoxycinnamic acid | C10H10O3 | 161.0591 | 351 | 3.9 | POS | 161.1;133.1;105.1 |
| Syringaldehyde | C9H10O4 | 183.0645 | 272 | 4.1 | POS | 123;95;155.1 |
| Uridine | C9H12N2O6 | 245.0762 | 79 | 2.6 | POS | 113;97;57 |
| Isocaproic acid | C6H12O2 | 115.0765 | 363.1 | 0.4 | NEG | 115.1;71;99.9 |
| 2',4'-Dihydroxyacetophenone | C8H8O3 | 151.0401 | 313.3 | 0.3 | NEG | 151;109;135 |
| (E)-9,12,13-trihydroxyoctadec-10-enoic acid | C18H34O5 | 329.2333 | 391.6 | 0.1 | NEG | 329.2;211.1;229.1 |
| (2R,3S,4S,5R,6R)-2-(hydroxymethyl)-6-[(2S,3R,4S,5S,6R)-3,4,5-trihydroxy-6-(hydroxymethyl)tetrahydropyran-2-yl]oxy-tetrahydropyran-3,4,5-triol | C12H22O11 | 341.1085 | 48.2 | 1.2 | NEG | 89;59;71 |
| 2-phenyl-2-[(2R,3R,4S,5S,6R)-3,4,5-trihydroxy-6-(hydroxymethyl)tetrahydropyran-2-yl]oxy-acetonitrile | C14H17NO6 | 294.0983 | 258.4 | 0.1 | NEG | 71;161;101 |
| (2R,3R,4S,5S,6R)-2-[(2E)-3,7-dimethylocta-2,6-dienoxy]-6-[[(2S,3R,4S,5R)-3,4,5-trihydroxytetrahydropyran-2-yl]oxymethyl]tetrahydropyran-3,4,5-triol | C21H36O10 | 447.2236 | 349.1 | 0.1 | NEG | 447.2;315.2;101 |
| 2-Methylcaproic acid | C7H14O2 | 129.0921 | 398.8 | 0.5 | NEG | 129.1;128;85 |
| (2R,3S,4S,5R,6R)-2-[[(2R,3R,4R)-3,4-dihydroxy-4-(hydroxymethyl)oxolan-2-yl]oxymethyl]-6-[(1,7,7-trimethyl-2-bicyclo[2.2.1]heptanyl)oxy]oxane-3,4,5-triol | C21H36O10 | 447.2239 | 337.2 | 0.9 | NEG | 447.2;315.2;101 |
| Methyl Paraben | C8H8O3 | 151.0401 | 326.6 | 0.1 | NEG | 151;93;136 |
| 2-phenyl-2-[3,4,5-trihydroxy-6-[[3,4,5-trihydroxy-6-(hydroxymethyl)oxan-2-yl]oxymethyl]oxan-2-yl]oxyacetic acid | C20H28O13 | 475.1456 | 207.8 | 0.2 | NEG | 101;71;59 |
| Vidarabine | C10H13N5O4 | 266.0895 | 182.7 | 0.3 | NEG | 134;266.1;135.1 |
| L-Uridine | C9H12N2O6 | 243.0622 | 79.4 | 0 | NEG | 110;200.1;152 |
| Citric acid | C6H8O7 | 173.0091 | 62.2 | 0.2 | NEG | 111;85;67 |
| Gluconic acid | C6H12O7 | 195.0507 | 42.2 | 1.4 | NEG | 195.1;75;129 |
| 1-beta-D-Arabinofuranosyluracil | C9H12N2O6 | 245.0762 | 79 | 2.6 | POS | 113;97;57 |
| Curdione | C15H24O2 | 237.1841 | 424.1 | 3.6 | POS | 135.1;237.2;219.2 |
| Maltose | C12H22O11 | 365.1044 | 45.2 | 3 | POS | 365.1;203.1;185 |
| Melibiose | C12H22O11 | 365.1044 | 45.2 | 3 | POS | 365.1;203.1;185 |
| 4-Guanidinobutyric acid | C5H11N3O2 | 146.0919 | 48.5 | 3.7 | POS | 146.1;87;86.1 |
| 3-Epioleanolic acid | C30H48O3 | 455.3528 | 534.1 | 0.5 | NEG | 455.4;50.6;454.3 |
| Quercetin 3-gentiobioside | C27H30O17 | 625.1419 | 275.5 | 1.5 | NEG | 300;625.1;301 |
| Isocitric acid | C6H8O7 | 173.0091 | 62.2 | 0.2 | NEG | 111;85;67 |
| Forskolin | C22H34O7 | 391.2126 | 402.4 | 0.2 | NEG | 391.2;319.2;347.2 |
| 2,3-bis[(4-hydroxy-3-methoxy-phenyl)methyl]butane-1,4-diol | C20H26O6 | 361.1653 | 305.4 | 0.9 | NEG | 361.2;199.1;155.1 |
| Phenol | C6H6O | 93.0346 | 300.4 | 0.1 | NEG | 93 |
| Kynurenic acid | C10H7NO3 | 188.0354 | 248.4 | 0.4 | NEG | 144;188;145 |
| Ethyl 4-hydroxybenzoate | C9H10O3 | 165.0557 | 362.7 | 0.2 | NEG | 165.1;150;122 |
| 1-phenylbutane-1,3-dione | C10H10O2 | 163.0747 | 455.7 | 4.1 | POS | 105;163.1;106 |
| (±)-Stylopine | C19H17NO4 | 324.1218 | 369.3 | 4 | POS | 324.1;147.1;250.1 |
| 1-(4-methoxyphenyl)ethanone | C9H10O2 | 151.0748 | 334 | 3.9 | POS | 151.1;43;123.1 |
| (S)-Scoulerine | C19H21NO4 | 328.1536 | 222.1 | 2.4 | POS | 297.1;265.1;328.2 |
| DL-Glutamic acid | C5H9NO4 | 130.0494 | 44.5 | 3.8 | POS | 84;130;84.1 |
| Glutamate | C5H9NO4 | 130.0494 | 44.5 | 3.8 | POS | 84;130;84.1 |
| Desaminotyrosine | C9H10O3 | 165.0557 | 280.5 | 0 | NEG | 150;165.1;151 |
| Palatinose (hydrate) | C12H22O11 | 341.1085 | 48.2 | 1.2 | NEG | 89;59;71 |
| Traumatic acid | C12H20O4 | 227.129 | 384.9 | 0.7 | NEG | 227.1;183.1;209.1 |
| (10E,15Z)-9,12,13-trihydroxyoctadeca-10,15-dienoic acid | C18H32O5 | 327.2179 | 375.9 | 0.7 | NEG | 327.2;211.1;171.1 |
| [(2R,3S,4S,5R,6S)-6-[[(1R,2S,3R,5R,6R,8S)-2-(benzoyloxymethyl)-6-hydroxy-8-methyl-9,10-dioxatetracyclo[4.3.1.02,5.03,8]decan-3-yl]oxy]-3,4,5-trihydroxy-tetrahydropyran-2-yl]methyl 4-hydroxybenzoate | C30H32O13 | 599.177 | 324.6 | 0.1 | NEG | 137;281.1;121 |
| (2R,3R,4S,5S,6R)-2-benzyloxy-6-[[(2S,3R,4S,5R)-3,4,5-trihydroxytetrahydropyran-2-yl]oxymethyl]tetrahydropyran-3,4,5-triol | C18H26O10 | 401.1454 | 247.1 | 0.4 | NEG | 269.1;101;401.1 |
| 3',4'-Dihydroxyacetophenone | C8H8O3 | 151.0401 | 245.9 | 0.4 | NEG | 151;108;109 |
| (2E,4E)-5-[8-hydroxy-1,5-dimethyl-3-[3,4,5-trihydroxy-6-(hydroxymethyl)tetrahydropyran-2-yl]oxy-6-oxabicyclo[3.2.1]octan-8-yl]-3-methyl-penta-2,4-dienoic acid | C21H32O10 | 443.1925 | 228.6 | 0.7 | NEG | 443.2;59;101 |
| Alisol B 23-acetate | C32H50O5 | 513.3584 | 512.7 | 0.2 | NEG | 513.4;57;451.3 |
| (1R,7R,10R)-4,10,11,11-tetramethyltricyclo[5.3.1.01,5]undec-4-en-3-one | C15H22O | 219.1736 | 458.8 | 3.4 | POS | 219.2;109.1;135.1 |
| [(2S,3R,4S,5S,6R)-3,4,5-trihydroxy-6-(hydroxymethyl)tetrahydropyran-2-yl] (E)-3-phenylprop-2-enoate | C15H18O7 | 293.1011 | 294.9 | 3 | POS | 131;274.1;292.1 |
| 2,6-Dimethoxy-1,4-benzoquinone | C8H8O4 | 151.0385 | 229.6 | 3.2 | POS | 151;123.1;95.1 |
| (2R)-3-(3,4-dihydroxyphenyl)-1-[2,4-dihydroxy-3-[(2S,3R,4R,5S,6R)-3,4,5-trihydroxy-6-(hydroxymethyl)tetrahydropyran-2-yl]phenyl]-2-hydroxy-propan-1-one | C21H24O11 | 453.1383 | 220.2 | 1.8 | POS | 123;163;452.2 |
| Corosolic acid | C30H48O4 | 471.3481 | 481.6 | 0.2 | NEG | 471.3;52.4;470.3 |
| Taxifolin | C15H12O7 | 303.0511 | 295.4 | 0.2 | NEG | 125;285;302 |
| Dihydroferulic acid | C10H12O4 | 195.0662 | 275.6 | 0.2 | NEG | 136.1;195.1;123 |
| Bayogenin | C30H48O5 | 487.3434 | 445.9 | 1.1 | NEG | 487.3;54.1;94.9 |
| D-(+)-Melibiose | C12H22O11 | 341.1085 | 48.2 | 1.2 | NEG | 89;59;71 |
| Phthalic acid | C8H6O4 | 165.0194 | 246.3 | 0.3 | NEG | 121;93;165 |
| (2R,3R,4S,5S,6R)-2-benzyloxy-6-[[(2R,3R,4R)-3,4-dihydroxy-4-(hydroxymethyl)tetrahydrofuran-2-yl]oxymethyl]tetrahydropyran-3,4,5-triol | C18H26O10 | 401.1454 | 247.1 | 0.4 | NEG | 269.1;101;401.1 |
| 5-(4-carboxy-3-methyl-butyl)-1,4a-dimethyl-6-methylene-decalin-1-carboxylic acid | C20H32O4 | 335.223 | 438.8 | 0.7 | NEG | 221.2;335.2;99.1 |
| (2S,3S)-3,5,7-trihydroxy-2-(4-hydroxyphenyl)chroman-4-one | C15H12O6 | 287.0563 | 316.3 | 0.7 | NEG | 259.1;125;287.1 |
| (2S)-2-(2,6-dihydroxyphenyl)-5,7-dihydroxy-chroman-4-one | C15H12O6 | 287.0563 | 316.3 | 0.7 | NEG | 259.1;125;287.1 |
| 2-hydroxy-3-methoxy-benzaldehyde | C8H8O3 | 151.04 | 287.2 | 0.1 | NEG | 136;89;150 |
| Helicin | C13H16O7 | 283.0824 | 248.4 | 0.4 | NEG | 85;129;283.1 |
| 2-Isopropylmalic acid | C7H12O5 | 175.0612 | 220 | 0.3 | NEG | 115;175.1;113.1 |
| Gentianose | C18H32O16 | 503.1615 | 52.4 | 0.4 | NEG | 89;59;101 |
| Raffinose | C18H32O16 | 503.1615 | 52.4 | 0.4 | NEG | 89;59;101 |
| 2-Ketogluconic acid | C6H10O7 | 193.0353 | 43.4 | 0.1 | NEG | 103;59;192.1 |
| Xanthoxylin | C10H12O4 | 179.0697 | 385.1 | 3.4 | POS | 179.1;133.1;43 |
| 1-methyl-2,3,4,9-tetrahydro-1H-pyrido[3,4-b]indole-3-carboxylic acid | C13H14N2O2 | 231.1121 | 245.6 | 3 | POS | 158.1;188.1;214.1 |
| Guanine | C5H5N5O | 152.0563 | 73.7 | 3 | POS | 152.1;97.1;43 |
| Uracil | C4H4N2O2 | 113.0341 | 49.4 | 3.9 | POS | 113;70;96 |
| Choline | C5H14NO | 104.1065 | 38.8 | 4.7 | POS | 104.1;60.1 |
| (1S,2R,4aS,6aS,6bR,10S,11R,12aR,14bS)-10,11-dihydroxy-1,2,6a,6b,9,9,12a-heptamethyl-2,3,4,5,6,6a,7,8,8a,10,11,12,13,14b-tetradecahydro-1H-picene-4a-carboxylic acid | C30H48O4 | 471.3481 | 481.6 | 0.2 | NEG | 471.3;52.4;470.3 |
| (1S,2R,4aS,6aS,6bR,10R,11R,12aR,14bS)-10,11-dihydroxy-1,2,6a,6b,9,9,12a-heptamethyl-2,3,4,5,6,6a,7,8,8a,10,11,12,13,14b-tetradecahydro-1H-picene-4a-carboxylic acid | C30H48O4 | 471.3481 | 481.6 | 0.2 | NEG | 471.3;52.4;470.3 |
| 17-[5-hydroxy-6-(1-hydroxy-1-methyl-ethyl)tetrahydropyran-3-yl]-4,4,10,13,14-pentamethyl-1,2,5,6,9,11,12,15,16,17-decahydrocyclopenta[a]phenanthren-3-one | C30H48O4 | 471.3481 | 481.6 | 0.2 | NEG | 471.3;52.4;470.3 |
| 17-ODYA | C18H32O2 | 279.2327 | 547.4 | 0.9 | NEG | 279.2 |
| 1,10-dihydroxy-9-(hydroxymethyl)-1,2,6a,6b,9,12a-hexamethyl-2,3,4,5,6,6a,7,8,8a,10,11,12,13,14b-tetradecahydropicene-4a-carboxylic acid | C30H48O5 | 487.3434 | 445.9 | 1.1 | NEG | 487.3;54.1;94.9 |
| (Z)-6,9,10-trihydroxyoctadec-7-enoic acid | C18H34O5 | 329.2335 | 405 | 0.7 | NEG | 329.2;199.1;211.1 |
| 2,5-Dihydroxyacetophenone | C8H8O3 | 151.0401 | 313.3 | 0.3 | NEG | 151;109;135 |
| Isomaltose | C12H22O11 | 341.1085 | 48.2 | 1.2 | NEG | 89;59;71 |
| D-Panose | C18H32O16 | 503.1615 | 52.4 | 0.4 | NEG | 89;59;101 |
| Ellagic acid | C14H6O8 | 300.9989 | 496.1 | 0.1 | NEG | 217.1;285.1;301 |
| (1R,2R,5S,8R,14R,15R,16S)-16-hydroxy-8-isopropenyl-1,2,14,17,17-pentamethyl-pentacyclo[11.7.0.02,10.05,9.014,18]icosane-5,15-dicarboxylic acid | C30H46O5 | 485.3273 | 492.4 | 0.2 | NEG | 485.3;441.3;53.9 |
| (10E,12E)-9-hydroxyoctadeca-10,12-dienoic acid | C18H32O3 | 295.2279 | 466.1 | 0.2 | NEG | 295.2;277.2;209.2 |
| 2-[(2R,4aR,8R,8aR)-8-hydroxy-4a,8-dimethyl-decalin-2-yl]prop-2-enoic acid | C15H24O3 | 251.1653 | 415 | 0.4 | NEG | 251.2;57;207.2 |
| [(1S,4aS,6S,7R,7aS)-6-hydroxy-7-(hydroxymethyl)-4-[[(2R,3R,4S,5S,6R)-3,4,5-trihydroxy-6-[[(2R,3R,4S,5S,6R)-3,4,5-trihydroxy-6-(hydroxymethyl)tetrahydropyran-2-yl]oxymethyl]tetrahydropyran-2-yl]oxymethyl]-1,4a,5,6,7,7a-hexahydrocyclopenta[c]pyran-1-yl] 3-methylbutanoate | C27H44O16 | 605.2449 | 270.6 | 0.2 | NEG | 605.2;101;89 |
| 2-[[3,4-dihydroxy-4-(hydroxymethyl)tetrahydrofuran-2-yl]oxymethyl]-6-(5-hydroxy-1,7,7-trimethyl-norbornan-2-yl)oxy-tetrahydropyran-3,4,5-triol | C21H36O11 | 463.2185 | 262.4 | 0 | NEG | 463.2;331.2;161 |
| 2,2'-Iminodiacetic acid | C4H7NO4 | 132.0302 | 39.2 | 0.4 | NEG | 115;88;71 |
| α-Cyperone | C15H22O | 219.1736 | 458.8 | 3.4 | POS | 219.2;109.1;135.1 |
| (6S,7aR)-6-hydroxy-4,4,7a-trimethyl-6,7-dihydro-5H-benzofuran-2-one | C11H16O3 | 197.1166 | 296.6 | 3.3 | POS | 197.1;179.1;135.1 |
| (E)-3-(4-hydroxy-3-methoxy-phenyl)prop-2-enamide | C10H11NO3 | 176.07 | 248.7 | 3.2 | POS | 176.1;158.1;148.1 |
| 3,5-Dihydroxybenzoic acid | C7H6O4 | 153.0193 | 205.7 | 0.2 | NEG | 109;153;108 |
| 18α-Glycyrrhetinic acid | C30H46O4 | 469.3323 | 480.7 | 0 | NEG | 469.3;439.3;367.3 |
| 4-Ethoxybenzoic acid | C9H10O3 | 165.0557 | 362.7 | 0.2 | NEG | 165.1;150;122 |
| (4aS,6aR,6aS,6bR,8aR,10S,12aR,14bS)-10-hydroxy-1,2,6a,6b,9,9,12a-heptamethyl-4,5,6,6a,7,8,8a,10,11,12,13,14b-dodecahydro-3H-picene-4a-carboxylic acid | C30H46O3 | 453.3373 | 542.3 | 0.2 | NEG | 453.3;50.4;50.5 |
| Vanillic acid | C8H8O4 | 167.035 | 256.4 | 0.1 | NEG | 152;108;167 |
| Griffonilide | C8H8O4 | 167.035 | 179.2 | 0.1 | NEG | 167;123;93 |
| D-Phenylalanine | C9H11NO2 | 164.0717 | 143.5 | 0.2 | NEG | 147;164.1;72 |
| Benzoyloxypaeoniflorin | C30H32O13 | 583.1792 | 327.2 | 3.1 | POS | 105;385.1;151.1 |
| Eicosapentaenoic acid | C20H30O2 | 301.2173 | 539.9 | 0.1 | NEG | 301.2;301;79 |
| 4-Hydroxybenzyl alcohol | C7H8O2 | 123.0452 | 215.7 | 0.6 | NEG | 123;122;95 |
| 3-(3-Hydroxyphenyl)propanoic acid | C9H10O3 | 165.0557 | 310.4 | 0.1 | NEG | 121.1;165.1;147 |
| 2,4-bis(3-methylbut-2-enyl)-6a,11a-dihydro-6H-benzofuro[3,2-c]chromene-3,9-diol | C25H28O4 | 391.1892 | 466.1 | 5.8 | NEG | 391.2;79;97 |
| 3,4-O-Isopropylidene-shikimic acid | C10H14O5 | 195.0663 | 253.8 | 0.3 | NEG | 180;151.1;195.1 |
| Boldine | C19H21NO4 | 328.1536 | 222.1 | 2.4 | POS | 297.1;265.1;328.2 |
| LPC(16:0) | C24H50NO7P | 496.3379 | 509.9 | 3.7 | POS | 184.1;104.1;496.3 |
| Glu-Glu | C10H16N2O7 | 259.0917 | 40.6 | 3.1 | POS | 242.1;126.1;128.1 |
| Proline | C5H9NO2 | 116.0702 | 52.5 | 3.9 | POS | 70.1;116.1;71.1 |
| D-Proline | C5H9NO2 | 116.0702 | 52.5 | 3.9 | POS | 70.1;116.1;71.1 |
| 3-Methoxyphenylacetic acid | C9H10O3 | 165.0557 | 310.4 | 0.1 | NEG | 121.1;165.1;147 |
| 2-Phenyllactic acid | C9H10O3 | 165.0557 | 280.5 | 0 | NEG | 150;165.1;151 |
| 10,11-dihydroxy-9-(hydroxymethyl)-1,2,6a,6b,9,12a-hexamethyl-2,3,4,5,6,6a,7,8,8a,10,11,12,13,14b-tetradecahydro-1H-picene-4a-carboxylic acid | C30H48O5 | 487.3434 | 445.9 | 1.1 | NEG | 487.3;54.1;94.9 |
| Manninotriose | C18H32O16 | 503.1615 | 52.4 | 0.4 | NEG | 89;59;101 |
| 3,5-Di-tert-butylphenol | C14H22O | 205.1598 | 493.4 | 0.1 | NEG | 205.2;162.9 |
| Citramalic acid | C5H8O5 | 147.0298 | 61.4 | 0.4 | NEG | 147;87;129 |
| Palmitamide | C16H33NO | 256.2623 | 539.2 | 4.7 | POS | 256.3;88.1;57.1 |
| N-Acetylglutamic acid | C7H11NO5 | 190.0705 | 68.9 | 3 | POS | 130;144.1;148 |
| 2-Hydroxypalmitic acid | C16H32O3 | 271.2278 | 532.5 | 0.3 | NEG | 59;271.2;166.9 |
| Homovanillic alcohol | C9H12O3 | 149.0607 | 259.2 | 0 | NEG | 149.1;134;148.1 |
| Scopolin | C16H18O9 | 353.0877 | 246.3 | 0.1 | NEG | 173;191.1;179 |
| 3-methoxy-4-[3,4,5-trihydroxy-6-(hydroxymethyl)tetrahydropyran-2-yl]oxy-benzoic acid | C14H18O9 | 329.0882 | 214.3 | 1.1 | NEG | 167;89;59 |
| Monobutyl phthalate | C12H14O4 | 221.0819 | 385.9 | 0.2 | NEG | 71.1;121;69 |
| methyl 2-hydroxy-6-(4-hydroxy-2-methoxy-6-methoxycarbonyl-phenoxy)-4-methyl-benzoate | C18H18O8 | 361.0928 | 347.1 | 0.2 | NEG | 181.1;361.1;182.1 |
| 2-Hydroxy-6-methoxybenzoic acid | C8H8O4 | 167.0348 | 271 | 0.8 | NEG | 167;152;125 |
| Propyl paraben | C10H12O3 | 179.0711 | 361.8 | 1.3 | NEG | 59;134.1;71 |
| Iristectorin B | C23H24O12 | 493.1324 | 287.8 | 3.3 | POS | 153;123;331.1 |
| D-(+)-Cellobiose | C12H22O11 | 325.112 | 51.2 | 2.7 | POS | 308.1;280.1;165.1 |
| (2R,3R,4S,5S,6R)-2-benzyloxy-6-[[(2S,3R,4S,5S)-3,4,5-trihydroxytetrahydropyran-2-yl]oxymethyl]tetrahydropyran-3,4,5-triol | C18H26O10 | 401.1454 | 247.1 | 0.4 | NEG | 269.1;101;401.1 |
| (3R,4S,5S,6R)-6-[[(2S,3R,4S,5R,6R)-3,4,5-trihydroxy-6-[[(2S,3R,4S,5R,6R)-3,4,5-trihydroxy-6-(hydroxymethyl)tetrahydropyran-2-yl]oxymethyl]tetrahydropyran-2-yl]oxymethyl]tetrahydropyran-2,3,4,5-tetrol | C18H32O16 | 503.1615 | 52.4 | 0.4 | NEG | 89;59;101 |
| Rhein | C15H8O6 | 283.025 | 417.8 | 0.6 | NEG | 257;239;183 |
| Phenylacetic acid | C8H8O2 | 135.0451 | 292.5 | 0.1 | NEG | 135;93;120 |
| [(2R,3R,4S,5R,6S)-2-(hydroxymethyl)-4,5,6-tris[(3,4,5-trihydroxybenzoyl)oxy]tetrahydropyran-3-yl] 3,4,5-trihydroxybenzoate | C34H28O22 | 787.1006 | 279.7 | 0.8 | NEG | 169;787.1;617.1 |
| Stearamide | C18H37NO | 284.2935 | 564.9 | 4.6 | POS | 284.3;88.1;102.1 |
| Veratraldehyde | C9H10O3 | 167.0696 | 310.3 | 4.1 | POS | 43;167.1;125.1 |
| Corilagin | C27H22O18 | 633.074 | 232.8 | 1.1 | NEG | 301;633.1;275 |
| Magnolioside | C16H18O9 | 353.0877 | 246.3 | 0.1 | NEG | 173;191.1;179 |
| Phthalide | C8H6O2 | 135.0435 | 286.9 | 4.2 | POS | 135;95;79.1 |
| (E)-3-(2,5-dihydroxyphenyl)prop-2-enoic acid | C9H8O4 | 179.035 | 262.4 | 0.5 | NEG | 135;179;136 |
| methyl 2,4-dihydroxy-3,6-dimethyl-benzoate | C10H12O4 | 177.0557 | 361.8 | 0.5 | NEG | 149.1;177.1;134 |
| Nomilin | C28H34O9 | 495.2006 | 345.8 | 3.6 | NEG | 137;495.2;121 |
| Rutin | C27H30O16 | 609.1465 | 269.1 | 0.7 | NEG | 167;123;609.1 |
| Chlorogenic acid | C16H18O9 | 353.0878 | 220 | 0 | NEG | 191.1;179;135 |
| phenethanolamine | C8H11NO | 120.0803 | 97.2 | 3.6 | POS | 120.1;103.1;93.1 |
| 1,11-dihydroxy-1,2,6a,6b,9,9,12a-heptamethyl-10-oxo-3,4,5,6,6a,7,8,8a,11,12,13,14b-dodecahydro-2H-picene-4a-carboxylic acid | C30H46O5 | 467.3168 | 476.7 | 0.4 | NEG | 467.3;51.9 |
| (2R,3S,4S,5R,6R)-2-[[(2S,3R,4R)-3,4-dihydroxy-4-(hydroxymethyl)tetrahydrofuran-2-yl]oxymethyl]-6-(2-phenylethoxy)tetrahydropyran-3,4,5-triol | C19H28O10 | 415.161 | 276.4 | 0.2 | NEG | 99;89;59 |
| (S)-2-Hydroxy-3-phenylpropanoic acid | C9H10O3 | 165.0558 | 226.4 | 0.5 | NEG | 165.1;122;150 |
| 2-Hydroxyisobutyric acid | C4H8O3 | 85.0294 | 49.6 | 0.1 | NEG | 85;57;55 |
| Crotonoside | C10H13N5O5 | 284.0983 | 73.7 | 2.2 | POS | 152.1;153;70.1 |
| 1-[2,4-dihydroxy-6-[(2S,3R,4S,5S,6R)-3,4,5-trihydroxy-6-(hydroxymethyl)tetrahydropyran-2-yl]oxy-phenyl]ethanone | C14H18O9 | 329.0882 | 214.3 | 1.1 | NEG | 167;89;59 |
| methyl 2-hydroxy-3-[(2S,3R,4S,5S,6R)-3,4,5-trihydroxy-6-(hydroxymethyl)tetrahydropyran-2-yl]oxy-benzoate | C14H18O9 | 329.0881 | 230 | 0.8 | NEG | 209;167;269.1 |
| [(2R,3R,4R)-4-formyl-2,3,4-trihydroxy-5-(3,4,5-trihydroxybenzoyl)oxy-pentyl] 3,4,5-trihydroxybenzoate | C20H20O14 | 483.0781 | 207.4 | 0.2 | NEG | 169;483.1;331.1 |
| Maleic acid | C4H4O4 | 115.0036 | 59.9 | 0.2 | NEG | 71;115;114.1 |
| 2-Ketobutyric acid | C4H6O3 | 101.0244 | 51.6 | 0.3 | NEG | 73;55;101 |
| Helioscopinolide A | C20H28O3 | 317.21 | 423.7 | 3.6 | POS | 316.3;317.2;207.1 |
| 1-(1H-indol-3-yl)ethanone | C10H9NO | 160.0751 | 293.2 | 4.1 | POS | 160.1;119;105.1 |
| (5R,10S,13R,14R,17R)-17-[(1R,4S)-4,5-dihydroxy-1,5-dimethyl-hexyl]-4,4,10,13,14-pentamethyl-2,5,6,11,12,15,16,17-octahydro-1H-cyclopenta[a]phenanthrene-3,7-dione | C30H48O4 | 471.3481 | 481.6 | 0.2 | NEG | 471.3;52.4;470.3 |
| 9-hydroxy-7-isopropyl-1,4a-dimethyl-2,3,4,9,10,10a-hexahydrophenanthrene-1-carboxylic acid | C20H28O3 | 315.1967 | 431.4 | 0.5 | NEG | 315.2;300;314.2 |
| 2'-Hydroxy-5'-methylacetophenone | C9H10O2 | 149.0608 | 352.8 | 0.1 | NEG | 149.1;134;107.1 |
| Propionic acid | C3H6O2 | 73.0295 | 63.8 | 0.1 | NEG | 73;45;72 |
| (1R,9S,10S)-3,4-dihydroxy-5-isopropyl-11,11-dimethyl-16-oxatetracyclo[7.5.2.01,10.02,7]hexadeca-2,4,6-triene-8,15-dione | C20H24O5 | 343.1551 | 386.7 | 0 | NEG | 343.2;328.1;136 |
| Sec-O-Glucosylhamaudol | C21H26O10 | 437.1454 | 304.8 | 0.2 | NEG | 437.1;59;89 |
| Citraconic acid | C5H6O4 | 129.0193 | 61.8 | 0.2 | NEG | 128;85;129 |
| Oricinol | C7H8O2 | 123.0452 | 215.7 | 0.6 | NEG | 123;122;95 |
| Hydroxytyrosol acetate | C10H12O4 | 195.0662 | 275.6 | 0.2 | NEG | 136.1;195.1;123 |
| 1,3,5,6-tetrahydroxy-2-methyl-anthracene-9,10-dione | C15H10O6 | 285.0406 | 368.8 | 0.6 | NEG | 285;121;284.3 |
| (1R,2R,4aR,8aS)-1-[2-(3-furyl)ethyl]-2,4a,5-trimethyl-2,3,4,7,8,8a-hexahydronaphthalene-1-carboxylic acid | C20H28O3 | 315.1967 | 431.4 | 0.5 | NEG | 315.2;300;314.2 |
| 2'-Hydroxy-4'-methylacetophenone | C9H10O2 | 149.0608 | 307.1 | 0.1 | NEG | 149.1;131.1;43 |
| 4-Hydroxyphenylacetic acid | C8H8O3 | 151.0401 | 195 | 0.1 | NEG | 107.1;151;89 |
| Homogentisic acid | C8H8O4 | 149.0245 | 225.1 | 0.8 | NEG | 148.1;149;104.1 |
| [(2S,3R,4S,5S,6R)-3,4,5-trihydroxy-6-(hydroxymethyl)tetrahydropyran-2-yl] (2E,6E)-8-hydroxy-2,6-dimethyl-octa-2,6-dienoate | C16H26O8 | 345.1554 | 234.6 | 0.1 | NEG | 59;89;101 |
| 14-hydroxy-14-(hydroxymethyl)-5,9-dimethyl-tetracyclo[11.2.1.01,10.04,9]hexadecane-5-carboxylic acid | C20H32O4 | 335.223 | 438.8 | 0.7 | NEG | 221.2;335.2;99.1 |
| 1-Kestose | C18H32O16 | 503.1615 | 52.4 | 0.4 | NEG | 89;59;101 |
| Quillaic acid | C30H46O5 | 485.3273 | 492.4 | 0.2 | NEG | 485.3;441.3;53.9 |
| 2-Hydroxy-4-methoxybenzoic acid | C8H8O4 | 167.035 | 256.4 | 0.1 | NEG | 152;108;167 |
| 3-(4-Methoxyphenyl)propanoic acid | C10H12O3 | 179.0711 | 361.8 | 1.3 | NEG | 59;134.1;71 |
| Itaconic acid | C5H6O4 | 129.0193 | 61.8 | 0.2 | NEG | 128;85;129 |
| cis-​9,​10-​Epoxystearic acid | C18H34O3 | 297.2434 | 494.3 | 0.2 | NEG | 297.2;296.2;278.2 |
| 3-Hydroxydodecanoic acid | C12H24O3 | 215.1653 | 403.7 | 0.3 | NEG | 215.2;169.2;89 |
| 4-Chromanone | C9H8O2 | 147.045 | 357.1 | 1 | NEG | 147;103.1;104.1 |
| 3'-​O-​Acetylhamaudol | C17H18O6 | 317.1031 | 356.1 | 0.3 | NEG | 147;121;148 |
| 2-(1-carboxyethyl)-5-methyl-cyclopentanecarboxylic acid | C10H16O4 | 199.0977 | 342.8 | 0.5 | NEG | 155.1;199.1;156.1 |
| 3,4-Dihydrocoumarin | C9H8O2 | 147.0452 | 331.7 | 0.3 | NEG | 147;119.1;41 |
| Pyruvaldehyde | C3H4O2 | 71.0138 | 45.9 | 0.3 | NEG | 71;41;43 |
| Mesaconic acid | C5H6O4 | 129.0192 | 45.1 | 0.5 | NEG | 128;85;57 |
| 4-[1-hydroxy-2-(methylamino)ethyl]benzene-1,2-diol | C9H13NO3 | 164.0717 | 97.3 | 0.6 | NEG | 147;164.1;72 |
| Enterolactone | C18H18O4 | 297.1134 | 387.2 | 0.5 | NEG | 122;121;297.1 |
| (3Z)-3-butylidene-5-hydroxy-isobenzofuran-1-one | C12H12O3 | 203.0715 | 367 | 0.6 | NEG | 148;203.1;120 |
| 4-Hydroxyphenylacetaldehyde | C8H8O2 | 135.0452 | 281.4 | 0.4 | NEG | 135;93;120 |
| 2,6-Dihydroxybenzoic acid | C7H6O4 | 153.0193 | 242.8 | 0 | NEG | 153;151;125 |
| Kojic acid | C6H6O4 | 143.0334 | 64.9 | 3.3 | POS | 143;125;98.1 |
| 3-[6-[(2S,3R,4S,5S,6R)-3,4,5-trihydroxy-6-(hydroxymethyl)tetrahydropyran-2-yl]oxybenzofuran-5-yl]propanoic acid | C17H20O9 | 349.0929 | 321.4 | 0.1 | NEG | 109;195.1;261.1 |
| 3-[4-[(2S,3R,4S,5S,6R)-3,4,5-trihydroxy-6-(hydroxymethyl)tetrahydropyran-2-yl]oxyphenyl]propanoic acid | C15H20O8 | 327.1086 | 263.2 | 0.2 | NEG | 165.1;121.1;166.1 |
| (E,6R)-2,6-dimethyl-8-[(2R,3R,4S,5S,6R)-3,4,5-trihydroxy-6-(hydroxymethyl)tetrahydropyran-2-yl]oxy-oct-2-enoic acid | C16H28O8 | 347.1711 | 239.5 | 0.1 | NEG | 101;347.2;59 |
| Strychnine | C21H22N2O2 | 335.1707 | 209.8 | 14.1 | POS | 335.2;276.1;70.1 |
| 5-Hydroxyhexanoic acid | C6H12O3 | 131.0713 | 257.5 | 0 | NEG | 131.1;85.1;86.1 |
| Hamaudol | C15H16O5 | 275.0924 | 320.1 | 0.3 | NEG | 227.1;275.1;199.1 |
| 2-Hydroxyadenine | C5H5N5O | 152.0563 | 73.7 | 3 | POS | 152.1;97.1;43 |
| 2,2-dimethyl-6H-pyrano[3,2-c]quinolin-5-one | C14H13NO2 | 228.1012 | 340 | 3.1 | POS | 228.1;182.1;183.1 |
| 2-(1,3-benzodioxol-5-yl)-1-methyl-quinolin-4-one | C17H13NO3 | 280.096 | 297.5 | 3.2 | POS | 280.1;252.1;105.1 |
| Ilexgenin A | C30H46O6 | 483.3122 | 480.7 | 1.3 | NEG | 409.3;53.7;410.3 |
| 3,7-dihydroxy-9-methoxy-1-methyl-benzo[c]chromen-6-one | C15H12O5 | 271.0612 | 356.3 | 0.2 | NEG | 151;271.1;119.1 |
| (3S)-3-acetoxy-3-[(1R,2R,5R,6R,10S,11S,14S)-11-(3-furyl)-5-(1-hydroxy-1-methyl-ethyl)-2,6,10-trimethyl-3,13-dioxo-12,15-dioxatetracyclo[8.5.0.01,14.02,7]pentadecan-6-yl]propanoic acid | C28H36O10 | 513.2115 | 316.5 | 2.9 | NEG | 513.2;121;169 |
| (2R,3R,4S,5R,6R)-2-(hydroxymethyl)-6-[[(2R,3S,4S,5R,6S)-3,4,5-trihydroxy-6-[[(1S,2S,4S,5S,6R,10S)-5-hydroxy-2-(hydroxymethyl)-3,9-dioxatricyclo[4.4.0.02,4]dec-7-en-10-yl]oxy]tetrahydropyran-2-yl]methoxy]tetrahydropyran-3,4,5-triol | C21H32O15 | 505.1564 | 234.6 | 0.4 | NEG | 121;222.1;89 |
| 2-[2-hydroxy-2-(4-methoxyphenyl)-1-methyl-ethoxy]-6-(hydroxymethyl)tetrahydropyran-3,4,5-triol | C16H24O8 | 343.1398 | 210 | 0 | NEG | 59;89;71 |
| 3,7-Dimethyluric acid | C7H8N4O3 | 195.051 | 107.8 | 6.8 | NEG | 75;195.1;129 |
| N-Methylglutamic acid | C6H11NO4 | 142.0509 | 72.2 | 0.2 | NEG | 142.1;71;85 |
| Tyramine | C8H11NO | 120.0803 | 97.2 | 3.6 | POS | 120.1;103.1;93.1 |
| Mono(2-ethylhexyl) phthalate | C16H22O4 | 279.1581 | 472 | 3.6 | POS | 149;263.2;95.1 |
| 10-methoxy-2,2-dimethyl-pyrano[3,2-g]chromen-8-one | C15H14O4 | 259.0957 | 317.4 | 3.3 | POS | 127;218;114 |
| 4-Hydroxycinnamic acid | C9H8O3 | 147.0435 | 314.2 | 3.8 | POS | 147;103.1;91.1 |
| 2-Furoic acid | C5H4O3 | 111.0087 | 62.2 | 0.1 | NEG | 111;67;41 |
| 2-Hydroxyhexanedioic acid | C6H10O5 | 143.0349 | 51.6 | 0.2 | NEG | 71;85;143 |
| (-)-Gallocatechin | C15H14O7 | 289.0697 | 241.2 | 3.1 | POS | 123;163;139 |
| (+)-Gallocatechin | C15H14O7 | 289.0697 | 241.2 | 3.1 | POS | 123;163;139 |
| 2-Methoxybenzoic-acid | C8H8O3 | 135.0435 | 286.9 | 4 | POS | 135;95;79.1 |
| 2-Methylcyclopentane-1,3-dione | C6H8O2 | 113.0594 | 202.9 | 3.3 | POS | 113.1;95;43 |
| Glucose | C6H12O6 | 203.0519 | 103 | 3.9 | POS | 60;203.1;91.1 |
| 4-Aminobutyric acid (GABA) | C4H9NO2 | 86.0597 | 65.8 | 3.8 | POS | 85;86.1;57 |
| 3-Indoleethanol | C10H11NO | 162.0906 | 268.7 | 4.6 | POS | 161.1;162.1;162.1 |
| 4-methoxy-6-methyl-7,8-dihydro-5H-[1,3]dioxolo[4,5-g]isoquinoline | C12H15NO3 | 222.1119 | 149.1 | 2.9 | POS | 176.1;207;222.1 |
| 1-Caffeoylquinic acid | C16H18O9 | 353.0878 | 220 | 0 | NEG | 191.1;179;135 |
| DL-3-Phenyllactic acid | C9H10O3 | 147.0452 | 331.7 | 0.5 | NEG | 147;119.1;41 |
| NAE(18:2) | C20H37NO2 | 324.2885 | 517.5 | 3.8 | POS | 62.1;278.2;324.3 |
| Paeonol | C9H10O3 | 149.0593 | 97.2 | 3 | POS | 103.1;107;131 |
| 3-Hydroxyphenylacetic acid | C8H8O3 | 151.0401 | 195 | 0.1 | NEG | 107.1;151;89 |
| Flopropione | C9H10O4 | 181.0506 | 299.6 | 0.1 | NEG | 166;167;181.1 |
| Normetanephrine | C9H13NO3 | 166.0855 | 97.2 | 4.8 | POS | 120.1;166.1;121.1 |
| (2R,3R)-2-(2,6-dihydroxyphenyl)-3,5,7-trihydroxy-chroman-4-one | C15H12O7 | 303.0511 | 295.4 | 0.3 | NEG | 125;285;302 |
| 2-[(2R,4aR,5R,8S)-4a,8-dimethyl-5-[(2R,3R,4S,5S,6R)-3,4,5-trihydroxy-6-(hydroxymethyl)tetrahydropyran-2-yl]oxy-3,4,5,6,7,8-hexahydro-2H-naphthalen-2-yl]prop-2-enoic acid | C21H32O8 | 411.2026 | 400.3 | 0.5 | NEG | 411.2;59;231.1 |
| Lumichrome | C12H10N4O2 | 241.0718 | 264.1 | 5.5 | NEG | 241.1;124;169 |
| Benzyl butyl phthalate | C19H20O4 | 313.1424 | 415.4 | 3.4 | POS | 163.1;133.1;107 |
| 2,3-Dihydroxy-4-methoxyacetophenone | C9H10O4 | 183.0645 | 283.1 | 4.1 | POS | 183.1;43;141.1 |
| 5-[2-(3-furyl)ethyl]-8-hydroxy-5,6,8a-trimethyl-3,4,4a,6,7,8-hexahydronaphthalene-1-carboxylic acid | C20H28O4 | 331.1917 | 406.4 | 0.7 | NEG | 331.2;330.2;202.1 |
| Pseudolaric Acid B | C23H28O8 | 413.1639 | 401 | 8.3 | NEG | 413.2;59;97 |
| (12E)-16,18-dihydroxy-4-methyl-3-oxabicyclo[12.4.0]octadeca-1(14),12,15,17-tetraene-2,8-dione | C18H22O5 | 317.1429 | 422.4 | 11 | NEG | 317.1;97;169 |
| 5-(1,2,4a,5-tetramethyl-7-oxo-3,4,8,8a-tetrahydro-2H-naphthalen-1-yl)-3-methyl-pentanoic acid | C20H32O3 | 303.2308 | 412.1 | 3.3 | POS | 302.3;303.2;57.1 |
| 2-Aminoadipic acid | C6H11NO4 | 144.0651 | 76.8 | 2.8 | POS | 98.1;144.1;143 |
| Hypoxanthine | C5H4N4O | 135.0298 | 42.6 | 10.7 | NEG | 75;135;59 |
| Garcinone C | C23H26O7 | 413.1639 | 401 | 8.2 | NEG | 413.2;59;97 |
| 7-Methylxanthine | C6H6N4O2 | 165.0403 | 42.2 | 8.7 | NEG | 75;165;59 |
| 2-Methoxycinnamaldehyde | C10H10O2 | 163.0747 | 415.4 | 4.1 | POS | 55;163.1;107 |
| 5-hydroxy-3-(4-hydroxyphenyl)-7-[(2S,3R,4S,5S,6R)-3,4,5-trihydroxy-6-[[(2R,3R,4R,5R,6S)-3,4,5-trihydroxy-6-methyl-tetrahydropyran-2-yl]oxymethyl]tetrahydropyran-2-yl]oxy-chromen-4-one | C27H30O14 | 577.1482 | 250.5 | 13.9 | NEG | 407.1;289.1;125 |
| Inosine | C10H12N4O5 | 267.072 | 57.1 | 5.4 | NEG | 267.1;59;113 |
| 3-Methoxy-4-hydroxyphenylglycol sulfate | C9H12O7S | 263.0197 | 98.2 | 12.8 | NEG | 263;191;219 |
| Andrographolide | C20H30O5 | 351.2131 | 375.8 | 10.1 | POS | 351.2;222.1;306.1 |
| (3E,4R)-3-[2-[(1R,4aS,5R,6R,8aS)-6-hydroxy-5-(hydroxymethyl)-5,8a-dimethyl-2-methylene-decalin-1-yl]ethylidene]-4-hydroxy-tetrahydrofuran-2-one | C20H30O5 | 351.2131 | 375.8 | 10.1 | POS | 351.2;222.1;306.1 |
| Purine | C5H4N4 | 119.0349 | 52 | 11.7 | NEG | 59;71;89 |
| methyl (2S,4aR,6aR,7R,9S,10aS,10bR)-9-acetoxy-2-(3-furyl)-6a,10b-dimethyl-4,10-dioxo-2,4a,5,6,7,8,9,10a-octahydro-1H-benzo[f]isochromene-7-carboxylate | C23H28O8 | 413.1639 | 401 | 8.3 | NEG | 413.2;59;97 |
| Apigenin-7-O-rutinoside | C27H30O14 | 577.1482 | 250.5 | 13.9 | NEG | 407.1;289.1;125 |
| Rhoifolin | C27H30O14 | 577.1482 | 250.5 | 13.9 | NEG | 407.1;289.1;125 |
